# Supplementary material for: Non-Specific Signal Peptidase Processing of Extracellular Proteins in Staphylococcus aureus N315
Source: Proteomes. 2023 Feb 11;11(1):8. doi: 10.3390/proteomes11010008 (PMC9944065; doi:10.3390/proteomes11010008)
Supplement: Supplementary file 1 [file proteomes-11-00008-s001.zip › proteomes-2137845-supplementary.pdf]

Supplementary information

Non-specific signal peptidase processing during extracellular protein transport in *Staphylococcus aureus* N315

Santosh A. Misal[a,d]; Shital D. Ovhal[a], Sujun Li[b], Jonathan A. Karty[a], Haixu Tang[b], Predrag Radivojac[b,c], James P. Reilly[a §]

[a]Department of Chemistry, Indiana University, 800 E Kirkwood Avenue, Bloomington, IN 47405
[b]Luddy School of Informatics, Computing, and Engineering, 700 N. Woodlawn Avenue
Bloomington, IN 47408
[c]Khoury College of Computer Sciences, Northeastern University, 177 Huntington Avenue,
Boston, MA 02115
[d]Present address- Laboratory of Malaria, Immunology, and vaccinology, National Institute of Allergy and Infectious Diseases, National Institutes of Health, 9000 Rockville Pike, Bethesda MD 20892, USA. Email- santosh.misal@nih.gov

Supplementary Table S1. Signal peptide cleavage sites and middle cleavages in the protein sequence observed in top-down analysis agree with amidination bottom-up analysis.

No.	Protein accession/Name	Protein sequence in red color observed in the top-down analysis (Signal peptide ↓ cleavage site)	*N-term amidinated peptide observed in bottom-up analysis
1	P99134 Immunoglobulin G binding protein A precursor/ Spa, SACOL0095	MKKKNIYSIRKLGVGIASVTLGTLLISGGVTPAANA↓<br>**AQHDEAQQNAFYQVLNMPNLNADQR**NGFIQSLKDDPSQSANVLGEAQKLNDSQAPKADAQQNNFNK<br>DQQSAFYEILNMPNLNEAQRNGFIQSLKDDPSQSTNVLGEAKKLNESQAPKADNNFNKEQQNAFYEILN<br>MPNLNEEQRNGFIQSLKDDPSQSANLLSEAKKLNESQAPKADNKFNKEQQNAFYEILHLPNLNEEQRNGF<br>IQSLKDDPSVSKEILAEAKKLNDAQAPKEEDNKKPGKEDGNKPGKEDGNKPGKEDNKKPGKEDGNKPG<br>KEDNNKPGKEDGNKPGKEDNNKPGKEDGNKPGKEDGNKPGKEDGNGVHVVKPGDTVNDIAKANGTT<br>ADKIAADNKLADKNMIKPGQELVVDKKQPANHADANKAQALPET↓<br>**GEENPFIGTTVFGGLSLALGAALLAGRRREL**	***AQHDEAQQNAFYQVLNMPNLNADQR**<br><br>***GEENPFIGTTVFGGLSLALGAALLAGR<br>R**
2	Q99SU9 Staphylococcal complement inhibitor	**MKIRKSILAGTLAIVLASPLVTNLDKNEAQA**↓<br>**STSLPTSNEYQNEKLANELKSLLDELNVNELATGSLNTYYKRTIKISGLKAMYALKSKDFKKMSEA<br>KYQLQKIYNEIDEALKSKY**	***STSLPTSNEYQNEK**
3	P99160 Probable transglycosylase IsaA	**MKKTIMASSLAVALGVTGYAAGTGHQAHA**↓<br>**AEVNVDQAHLVDLAHNHQDQLNAAPIK**DGAYDIHFVKDGFQYNFTSNGTTWSWSYEAANGQTAGFS<br>NVAGADYTTSYNQGSDVQSVSYNAQSSNSNVEAVSAPTYHNYSTSTTSSSVRLSNGNTAGATGSSAAQI<br>MAQRTGVSASTWAAIIARESNGQVNAYNPSGASGLFQTMPGWGPTNTVDQQINAAVKAYKAQGLGAW<br>GF	***KKTIMASSLAVALGVTGYAAGTGHQA<br>HA**<br>***AEVNVDQAHLVDLAHNHQDQLNAAPI<br>K**
4		MKKTIMASSLAVALGVTGYAAGTGHQAHAAEVNVDQAHLVDLAHNHQDQLNAAPIKDGAYDIHFVKD<br>GFQYNFTSNGTTWSWSYEAANGQTAGFSNVAGADYTTSYNQGSDVQSVSYNAQSSNSNVEAVS↓<br>**APTYHNYSTSTTSSSVRLSNGNTAGATGSSAAQIMAQRTGVSASTWAAIIARESNGQVNAYNPSGAS<br>GLFQTMPGWGPTNTVDQQINAAVKAYKAQGLGAWGF**	***APTYHNYSTSTTSSSVR**
5	A0A0H3JK15 Uncharacterized protein	**MKKLLTASIIACSVVMGVGLVNTSAEA**↓<br>**ASGNSIDTVKQLIKGDQSLENVKIGESIKDVLTKYKNPMYSYNEDGTEHYYEFHTKKGMLLVTTDG<br>KKNNGKVTHISMMYNDANGPTYQAVKNYVGKAVTHTEYSKVAGNFGYIEKGKTTYQFASAPKDK<br>NIKLYRIDLEK**	***ASGNSIDTVK**
6	A0A0H3JPH2 Uncharacterized protein	**MKKKFVSSCIASTILFGTLLGVTYKAEA**↓<br>**ATVHVAGGVWSHGIGKHYVWSYYSHNKRNHGSTAVGKYSSFSGVARPGVQSKASAPKAWGGNK<br>TFYSLH**	***ATVHVAGGVWSHGIGK**<br>***ATVHVAGGVWSHGIGKHYVWSYYSH<br>NKR**
7		**MKKKFVSSCIASTILF**↓<br>**GTLLGVTYKAEAATVHVAGGVWSHGIGKHYVWSYYSHNKRNHGSTAVGKYSSFSGVARPGVQSK<br>ASAPKAWGGNKTFYSLH**	***GTLLGVTYK**

8		**MKKKFVSSCIAS**↓<br>**TILFGTLLGVTYKAEA**ATVHVAGGVWSHGIGKHYVWSYYSHNKRNHGSTAVGKYSSFSGVARPGVQS<br>KASAPKAWGGNKTFYSLH	***TILFGTLLGVTYK**
9		**MKKKFVSSCIASTILFGTLLGVTYKAEAATVHVAGGVWSHGIGKHYVWSYYSHNKRNHGSTAVG**<br>**KYSSF**↓**SGVARPGVQSKASAPKAWGGNKTFYSLH**	***SGVARPGVQSKASAPK**
10	A0A0H3JPQ1 SA1000 fibrinogen binding-related protein	**MKKNFIGKSILSIAAISLTVSTFAGESHA**↓<br>**QTKNVEAAKKYDQYQTNFKKQVNKKVVDAQKAVNLFKRTRTVATHRKAQRAVNLIHFQHSYEK**<br>**KKLQRQIDLVLKYNTLK**	* **QTKNVEAAKK**
11	P61598 Putative surface protein SA2285	MRDKKGPVNKRVDFLSNKLNKYSIRKFTVGTASILIGSLMYLGTQQEAEA↓<br>**AENNIENPTTLKDNVQSKEVK**IEEVTNKDTAPQGVEAKSEVTSNKDTIEHEASVKAEDISKKEDTPKEV<br>ANVAEVQPKSSVTHNAEAPKVRKARSVDEGSFDITRDSKNVVESTPITIQGKEHFEGYGSVDIQKNPTDL<br>GVSEVTRFNVGNESNGLIGALQLKNKIDFSKDFNFKVRVANNHQSNTTGADGWGFLFSKGNAEEYLTNG<br>GILGDKGLVNSGGFKIDTGYIYT↓<br>**SSMDKTEKQAGQGYRGYGAFVKNDSSGNSQMVGENIDKSKTNFLNYADNSTNTSDGKFHGQRLN**<br>**DVILTYV**ASTGKMRAEYAGKTWETSITDLGLSKNQAYNFLITSSQRWGLNQGINANGWMRTDLKGSEF<br>TFTPSAKNNNRIRKKVEEIPFKKERKFNPDLAPGTEKVTREGQKGEKTITTPTLKNPLTGEIISKGESKEEIT<br>KDPINELTEYGPETIAPGHRDEFDPKLPTGEKEEVPGKPGIKNPETGDVVRPPVDSVTKYGPVKGDSIVEK<br>EEIPFEKERKFNPDLAPGTEKVTREGQKGEKTITTPTLKNPLTGEIISKGESKEEITKDPINELTEYGPETIAP<br>GHRDEFDPKLPTGEKEEVPGKPGIKNPETGDVVRPPVDSVTKYGPVKGDSIVEKEEIPFEKERKFNPDLAP<br>GTEKVTREGQKGEKTITTPTLKNPLTGEIISKGESKEEITKDPINELTEYGPETIAPGHRDEFDPKLPTGEKE<br>EVPGKPGIKNPETGDVVRPPVDSVTKYGPVKGDSIVEKEEIPFEKERKFNPDLAPGTEKVTREGQKGEKTI<br>TTPTLKNPLTGEIISKGESKEEITKDPINELTEYGPETIAPGHRDEFDPKLPTGEKEEVPGKPGIKNPETGDV<br>VRPPVDSVTKYGPVKGDSIVEKEEIPFKKERKFNPDLAPGTEKVTREGQKGEKTITTPTLKNPLTGEIISKG<br>ESKEEITKDPINELTEYGPETITPGHRDEFDPKLPTGEKEEVPGKPGIKNPETGDVVRPPVDSVTKYGPVKG<br>DSIVEKEEIPFEKERKFNPDLAPGTEKVTREGQKGEKTITTPTLKNPLTGEIISKGESKEEITKDPVNELTEF<br>GGEKIPQGHKDIFDPNLPTDQTEKVPGKPGIKNPDTGKVIEEPVDDVIKHGPKTGTPETKTVEIPFETKREF<br>NPKLQPGEERVKQEGQPGSKTITTPITVNPLTGEKVGEGQPTEEITKQPVDKIVEFGGEKPKDPKGPENPE<br>KPSRPTHPSGPVNPNNPGLSKDRAKPNGPVHSMDKNDKVKKSKIAKESVANQEKKRAELPKTGLESTQK<br>GLIFSSIIGIAGLMLLARRRKN	***AENNIENPTTLKDNVQSKEVK**<br><br>***SSMDKTEKQAGQGYR**
12	A0A0H3JLJ8 SA0914, chitinase-related protein	**MNKLLQSLSALGVSATLVTPNLNADA**↓<br>**TTNTTPQIKGANDIVIKKGQDYNLLNGISAFDKEDGDLTDKIKVDGQIDTSKSGKYQIKYHVTDSDG**<br>**AIKISTRYIEVK**	***TTNTTPQIKGANDIVIKK**
13	A0A0H3JLH5 Uncharacterized protein SA0395	**MKFKKVLVATAMVGVLATGVVGYGNQADA**↓<br>**KVYSQNGLVLHDDANFLEHELSYIDVLLDKNADQATKDNLRSYFADKGLHSIKDIINKAKQDGFDV**<br>**SKYEHVK**	***KVYSQNGLVLHDDANFLEHELSYIDVL**<br>**LDK**

14	Q99TJ8 SACOL1710 Valine-tRNA ligase valS	MEMKPKYDPREVEAGRYEEWVKNGYFKPSEDKSKETYTIVIPPPNVTGKLHLGHAWDTTLQDIITRMKR<br>MQGYDTLYLPGMDHAGIATQAKVEAKLNEQGITRYDLGREKFLEQAWDWKEEYASFIRAQWAKLGLG<br>LDYSRERFTLDEGLSKAVKKVFVDLYNKGIIYRGERIINWDPKARTALSDIEVIHEDVQGAFYHFKYPYA<br>DGEGFIEIATTRPETMLGDTAIVVNPNDERYKDVIGKTVILPIVGRELPILADEYVDIDFGSGAMKVTPAH<br>DPNDFEIGQRHQLENIIVMDENGKMNDKAGKYEGMDRFDCRKQLVKDLKEQDLVIKIEDHVHSVGHSE<br>RSGAVVEPYLSTQWFVRMEDLAKRSLDNQKTDDRIDFYPQRFEHTFNQWMENIRDWTISRQLWWGHQI<br>PAWYHKETGEIYVGEEAPTDIENWQQDEDVLDTWFSSALWPFSTLGWPDLESEDFKRYYPTNALVTGY<br>DIIFFWVARMIFQGLEFTDRRPFNDVLLHGLVRAEDGRKMSKSLGNGVDPMDVIDEYGADSLRYFLATG<br>SSPGHDLRYSTEKVESVWNFINKIWNGARFSLMNIGEDFKVEDIDLSGNLSLADKWILTRLNETIATVTDL<br>SDKYEFGEVGRALYNFIWDDFCDWYIEMSKIPMNSNDEEQKQVTRSVLSYTLDNIMRMLHPFMPFVTEK<br>IWQSLPHEGDTIVKASWPEVRESLIFEESKQTMQQLVEIIKSVRQSRVEVNTPLSKEIPILIQAKDKEIETTLS<br>QNKDYLIKFCNPSTLNISTDVEIPEKAMTSVVIAGKVVLPLEGLIDMDKEISRLEK↓<br>**ELAKLQSELDRVDK**KLSNENFVSKAPEKVINEEKRKKQDYQEKYDGVKARIEQLKA	* **ELAKLQSELDRVDK**
15	A0A0H3JM99 SA1477, Uncharacterized protein	MSILT↓**IILIALLVILLF**RVGLSILRFLIYVGLVLLCIYLGYQGLIWLLDFFQINSGFLPHFQFNN	***IILIALLVILLFRVGLSILR**
16	A0A0H3JLW4 SA1235, conserved hypothetical protein	**MWTVTKIRADYEGWWLFSDW**↓<br>**PENIVEKYQYQDFDDMFKHYQQLIN**QCKVQFDNYVTGKYNIYAFYNNCDMNYCEDCEEDLQIFYSFI<br>VLQNNEVYYKLPIID	***PENIVEKYQYQDFDDMFKHYQQLINQ CKVQFDNYVTGK**
17	P65986 DNA repair protein RecO	MLMRQKGIIIKAVDYGESDKIITILNEHGAKVPLMARRAKKVKTGLQAQTQLFVYGLFIYNQWRGMGTL<br>NSVDVISQHYKLQMDLYVSSYASLAAETIERSMDEGDIAPYNYQLLQFVLEKIESGTSAQLMSVVVMLK<br>CMKRFGFTASFNRCAVSGNDTQADLIGYSFKFDGAISRQEASK↓<br>**DVHAVILSNKTLYLLDVLQKLPIDKMNS**LNIHQEIIDEMSDIILMLYREYAGMFFKSQKLINQLKRLEQ	***DVHAVILSNK**

Supplementary Table S2: Alternate signal peptide cleavages occurred during the SPase processing.

	**Protein accession, name, and sequence**
**1**	**Q7A5M1 Extracellular matrix-binding protein**<br><br>MNYRDKIQKFSIRKYTVGTFSTVIATLVFLGFNTSQAH↓**A**↓**A**↓**E**↓**TNQPASVVKQKQQSNNEQTENR**ESQVQNSQNSQNSQSLSATHENEQPNNSQANL<br>VNQKVAQSSTTNDEQ^{160}... ^{3870}LGTLTHITTAQRNDLTNQIS<br><br>Additional cleavages at one or more residues (↓) on either side of AXA<br>Known/predicted signal peptide cleavage (↓) follows AXA pattern
**2**	**P99134 Immunoglobulin G binding protein A**<br><br>MKKKNIYSIRKLGVGIASVTLGTLLISGGVTPAAN↓**A**↓**A**↓**QHDEAQQNAFYQVLNMPNLNADQR**NGFIQSLKDDPSQSANVLGEAQKLNDSQAPKADAQQ<br>NNFNKDQQSAFYEILNMPNLNEAQRNGFIQSLKDDPSQSTNVLGEAKKLNESQAPKADNNFNKEQQNAFYEILNMPNLNEEQRNGFIQSLKDDPSQSANLL<br>SEAKKLNESQAPKADNKFNKEQQNAFYEILHLPNLNEEQRNGFIQSLKDDPSVSKEILAEAKKLNDAQAPKEEDNKKPGKEDGNKPGKEDGNKPGKEDNKK<br>PGKEDGNKPGKEDNNKPGKEDGNKPGKEDNNKPGKEDGNKPGKEDGNKPGKEDGNGVHVVKPGDTVNDIAKANGTTADKIAADNKLADKNMIKPGQELVVD<br>KKQPANHADANKAQALPET↓**GEENPFIGTTVFGGLSLALGAALLAGR**RREL
**3**	**P61598 Putative surface protein SA2285**<br><br>MRDKKGPVNKRVDFLSNKLNKYSIRKFTVGTASILIGSLMYLGTQQEAEA↓**A**↓**ENNIENPTTLKDNVQSK**EVKIEEVTNKDTAPQGVEAKSEVTSNKDTI<br>EHEASVKAEDISKKEDTPKEVANVAEVQPKSSVTHNAEAPKVRKARSVDEGSFDITRDSKNVVESTPITIQGKEHFEGYGSV^{180}......^{1281}ENPEKPSRPTHPS<br>GPVNPNNPGLSKDRAKPNGPVHSMDKNDKVKKSKIAKESVANQEKKRAELPKTGLESTQKGLIFSSIIGIAGLMLLARRRKN
**4**	**A0A0H3JNG8 Staphylocoagulase**<br><br>MKKQIISLGALAVASSLFTWDNKADA↓**I**↓**VTKDYSKESRVNEK**SKKGATVSDYYYWKIIDSLEAQFTGAIDLLEDYKYGDPIYKEAKDRLMTRVLGEDQY<br>LLKKKIDEYELYKKWYKSSNKNTNMLTFHKYNLYNLTMNEYNDIFNSLKDAVYQFNKEVKEIEHKNVDLKQFDKDGEDKATKEVYDLVSEIDTLVVTYYAD<br>KDYGEHAKELRAKLDLILGDTDNPHKITNERIKKEMIDDLNSIIDDFFMETKQNRPNSITKYDPTKHNFKEKSENKPNFDKLVEETKKAVKEADESWKNKT<br>VKKYEETVTKSPVVKEEKKVEEPQLPKVGNQQEVKTTAGKAEETTQPVAQPLVKIPQETIYGETVKGPEYPTMENKTLQGEIVQGPDFLTMEQNRPSLSDN<br>YTQPTTPNPILEGLEGSSSKLEIKPQGTESTLKGIQGESSDIEVKPQATETTEASQYGPRPQFNKTPKYVKYRDAGTGIREYNDGTFGYEARPRFNKPSET<br>NAYNVTTNQDGTVSYGARPTQNKPSETNAYNVTTHANGQVSYGARPTQKKPSKTNAYNVTTHANGQVSYGARPTQKKPSKTNAYNVTTHANGQVSYGARPT<br>YKKPSETNAYNVTTHANGQVSYGARPTQKKPSETNAYNVTTHADGTATYGPRVTK
**5**	**Q7A4V3 UPF0342 protein SA1663**<br><br>MAVNLYDYANQLEQALRESEEYKAIKEAF↓**AN**↓**VKANEESKK**LFDEFRETQINFQQKQMQGEEIAEEDLQKAQEQAQAIEKDENISALMNAEQKMSQVFQ<br>EINQIIVKPLDEIYAD

**6**	**Q99SU9 Staphylococcal complement inhibitor**<br><br>MKIRKSILAGTLAIVLASPLVTNLDKNEAQA↓**S**↓**TSLPTSNEYQNEK**LANELKSLLDELNVNELATGSLNTYYKRTIKISGLKAMYALKSKDFKKMSEAK<br>YQLQKIYNEIDEALKSKY
**7**	**Q7A6A6 Glutamyl endopeptidase**<br><br>MKGKFLKVSSLFVATLTTATLVSSPAANA↓**L**↓**SSKAMDNHPQQTQSSK**QQTPKIKKGGNLKPLEQREHANVILPNNDRHQITDTTNGHYAPVTYIQVEAP<br>TGTFIASGVVVGKDTLLTNKHVVDATHGDPHALKAFPSAINQDNYPNGGFTAEQITKYSGEGDLAIVKFSPNEQNKHIGEVVKPATMSNNAETQVNQNITV<br>TGYPGDKPVATMWESKGKITYLKGEAMQYDLSTTGGNSGSPVFNEKNEVIGIHWGGVPNEFNGAVFINENVRNFLKQNIEDIHFANDDQPNNPDNPDNPNN<br>PDNPNNPDNPNNPDEPNNPDNPNNPDNPDNGDNNNSDNPDAA

The Yellow highlighted sequence is the typical signal peptide with↓ a SPase cleavage site. The red arrow ↓ indicates the new additional cleavages.

A[160]… [3870]B- The superscript number denotes the residue number in the protein sequence. The longer protein sequence is omitted due to the limited space.

Supplementary Table S3: The non-specific cleavages occurred middle of the protein sequence in addition to the signal peptide cleavage during the SPase processing.

NO.	**Protein accession, name and sequence**
**1**	**A0A0H3JPH2, Uncharacterized protein**<br>MKKKFVSSCIAS↓TILF↓GTLLGVTYKAEA↓<br>**ATVHVAGGVWSHGIGKHYVWSYYSHNKR**NH**GSTAVGKYSSF**↓**SGVAR**PGVQSKASAPKAWGGNKTFYSLH
**2**	**P99160, Probable transglycosylase IsaA**<br>MKKTIMASSLAVALGVTGYAAGTGHQAHA↓<br>A**EVNVDQAHLVDLAHNHQDQLNAAPIK**DGAYDIHFVKDGFQYNFTSNGTTWSWSYEAANGQTAGFSNVAGADYTTSYNQGSDVQSVSYNAQSSN<br>SNVEAVS↓**APTYHNYSTSTTSSSVR**LSNGNTAGATGSSAAQIMAQRTGVSASTWAAIIARESNGQVNAYNPSGASGLFQTMPGWGPTNTVDQQ<br>INAAVKAYKAQGLGAWGF
**3**	**P68800, Fibrinogen-binding protein**<br>MKNKLIAKSLLTIAAIGITTTTIASTADA↓<br>**SEGYGPREKK**PVSINHN↓IVEYNDGTFKYQSRPKFNSTPKYIKFKHDYNILEFNDGTFEYGARPQFNKPAAKTDATIKKEQKLIQAQNLVREF<br>EKTHTVSAHRKAQKAVNLVSFEYKVKKMVLQERIDNV↓LKQGLVR
**4**	**P65289, Lipase 1**<br>MKSQNKYSIRKFSVGASSILIATLLFLSGGQAQA↓<br>AEKQVNMGNSQEDTVTAQSIGDQQTRENANYQRENGVDEQQHTENLTKNLHNDKTISEENHRKTDDLNKDQLKDDKNSSLNNKNIQRDTTKNNN<br>ANPSDVNQGLEQAINDGKQSKVASQQQSKEVDNSQDSNANNNLPSQSLTKEAPSLNKSDQTSQREIVNETEIEKVQPQQNNQANDKITNHNFNN<br>EQEVKPQKDEKTLSVSDLKNNQKSPVEPTKDNDKKNGLNLLKSSAVATLPNKGTKELTAKAKDDQTNKVAKQGQYKNQDPIVLVHGFNGFTDDI<br>NPSVLAHYWGGNKMNIRQDLEENGYKAYEASISAFGSNYDRAVELYYYIKGGRVDYGAAHAAKYGHERYGKTYEGIYKDWKPGQKVHLVGHSMG<br>GQTIRQLEELLRNGNREEIEYQKKHGGEISPLFKGNNDNMISSITTLGTPHNGTHASDLAGNEALVRQIVFDIGKMFGNKNSRVDFGLAQWGLK<br>QKPNESYIDYVKRVKQSNLWKSKDNGFYDLTREGATDLNRKTSL↓NPNIVYKTYTGEATHKALNSDRQKADLNMFFPFVITGNLIGKATEKEW<br>RENDGLVSVISSQHPFNQAYTNATDKIQKGIWQVTPTKHDWDHVDFVGQDSSDTVRTREELQDFWHHLADDLVKTEKVTDTKQA
**5**	**Q7A7P2, Lipase 2**<br>MLRGQEERKYSIRKYSIGVVSVLAATMFVVSSHEAQA↓<br>SEKTPTSNAAAQKETLNQPGEQGNAITSHQMQSGKQLDDMHKENGKSGTVTEGKDTLQSSKHQSTQNSKTIRTQNDNQVKQDSERQGSKQSHQN<br>NATNNTERQNDQVQNTHHAERNGSQSTTSQSNDVDKSQPSIPAQKVLPNHDKAAPTSTTPPSNDKTAPKSTKAQDATTDKHPNQQDTHQPAHQI<br>IDAKQDDTVRQSEQKPQVGDLSKHIDGQNSPEKPTDKNTDNKQLIKDALQAPKTRSTTNAAADAKKVRPLKANQVQPLNKYPVVFVHGFLGLVG<br>DNAPALYPNYWGGNKFKVIEELRKQGYNVHQASVSAFGSNYDRAVELYYYIKGGRVDYGAAHAAKYGHERYGKTYKGIMPNWEPGKKVHLVGHS<br>MGGQTIRLMEEFLRNGNKEEIAYHKAHGGEISPLFTGGHNNMVASITTLATPHNGSQAADKFGNTEAVRKIMFALNRFMGNKYSNIDLGLTQWG

	FKQLPNESYIDYIKRVSKSKIWTSDDNAAYDLTLDGSAKLNNMTSMNPNITYTTYTGVSSHTGPLGYENPDLGTFFLMDTTSRIIGHDAREEWR<br>KNDGVVPVISSLHPSNQPFINVTNDEPATRRGIWQVKPIIQGWDHVDFIGVDFLDFKRKGAELANFYTG↓**IINDLLR**VEATESKGTQLKAS
**6**	**Q99RL2, Immunoglobulin-binding protein Sbi**<br>MKNKYISKLLVGAATITLATMISNGEAKA↓<br>**SENTQQTSTK**HQTTQNNYVTDQQKAFYQVLH↓LKGITEEQRNQYIKTLREHPERAQEVFSESLKDSKNPDRRVAQQNAFYNVLKNDNLTEQEK<br>NNYIAQIKENPDRSQQVWVESVQSSKAKERQNIENADKAIKDFQDNKAPHDKSAAYEANSKLPKDLRDKNNRFVEKVSIEKAIVRHDERVKSAN<br>DAISKLNEKDSIENRRLAQREVNKAPMDVKEHLQKQLDALVAQKDAEKKVAPKVEAPQIQSPQIEKPKAESPKVEVPQIQSPKVEVPQSKLLGY<br>YQSLKDSFNYGYKYLTDTYKSYKEKYDTAKYYYNTYYKYKGAIDQTVLTVLGSGSKSYIQPLKVDDKNGYLAKSYAQVRNYVTESINTGKVLYT<br>FYQNPTLVKTAIKAQETASSIKNTLSNLLSFWK
**7**	**Q7A6A6 Glutamyl endopeptidase**<br>MKGKFLKVSSLFVATLTTATLVSSPAANA↓L↓SSKAMDNHPQQTQSSKQQTPKIKKGGN↓LKPLEQREHANVILPNNDRHQITDTTNGHYAP<br>VTYIQVEAPTGTFIASGVVVGKDTLLTNKHVVDATHGDPHALKAFPSAINQDNYPNGGFTAEQITKYSGEGDLAIVKFSPNEQNKHIGEVVKPA<br>TMSNNAETQVNQNITVTGYPGDKPVATMWESKGKITYLKGEAMQYDLSTTGGNSGSPVFNEKNEVIGIHWGGVPNEFNGAVFINENVRNFLKQN<br>IEDIHFANDDQPNNPDNPDNPNNPDNPNNPDNPNNPDEPNNPDNPNNPDNPDNGDNNNSDNPDAA
**8**	**A0A0H3JP98, SA0743 protein**<br>MKNKLLVLSLGALCVSQIWESNHASA↓<br>VVSGEKNPYVSKALELKDKSNKSNSYENYRDSLESLISSLSFADYEKYEEPEYEKAVKKYQQKFMAEDDALKNFLNEEKKIKNADISRKSNNLL<br>GLTHERYSYIFDTLKKNKQEFLKDIEEIQLKNSDLKDFNNTEQHNADVEINNLENKVLMVGYTFYNTNKDEVEELYSELDLIVGEVQDKSDKKR<br>AVNQRMLNRKKEDLEFIIDKFFKKIQQERPESIPALTSEKNHNQTMALKLKADTEAAKNDVSKRSKRSLNTQNNKSTTQEISEEQKAEYQRKSE<br>ALKERFINRQKSKNESVVSLIDDEDDNENDRQLVVS↓APSKKPTTPTTYTETTTQVPMPTVERQTQQQIVYKTPKPLAGLNGESHDFTTTHQS<br>PTTSNHTHNNVVEFEETSALPGRKSGSLVGISQIDSSHLTEREKRVIKREHVREAQKLVDNYKDTHSYKDRLNAQQKVNTLSEGHQKRFNKQIN<br>KVYNGK
**9**	**P61598, Putative surface protein SA2285**<br>MRDKKGPVNKRVDFLSNKLNKYSIRKFTVGTASILIGSLMYLGTQQEAEA↓<br>A↓ENNIENPTTLKDNVQSKEVKIEEVTNKDTAPQGVEAKS↓EVTSNKDTIEHEASVKAEDISKKEDTPKEVANVAEVQPKSSVTHNAEAPKV<br>RKARSVDEGSFDITRDSKNVVESTPITIQGKEHFEGYG↓SVDIQKNPTDLGVSEVTRFNVGNESNGLIGALQLKNKIDFSKDFNFKVRVANNH<br>QSNTTGADGWGFLFSKGNAEEYLTNGGILGDKGLVNSGGFKIDTGYIYT↓**SSMDKTEKQAGQGYR**GYGAFVKNDSSGNSQMVGENIDKSKTNF<br>LNYADNSTNTSDGKFHGQRLNDVILTYVASTGKMRAEYAGKTWETSITDLGLSKNQAYNFLITSSQRWGLNQGINANGWMRTDLKGSEFTFTPS<br>AKNNNRIRKKVEEIPFKKERKFNPDLAPGTEKVTREGQKGEKTITTPTLKNPLTGEIISKGESKEEITKDPINELTEYGPETIAPGHRDEFDPK<br>LPTGEKEEVPGKPGIKNPETGDVVRPPVDSVTKYGPVKGDSIVEKEEIPFEKERKFNPDLAPGTEKVTREGQKGEKTITTPTLKNPLTGEIISK<br>GESKEEITKDPINELTEYGPETIAPGHRDEFDPKLPTGEKEEVPGKPGIKNPETGDVVRPPVDSVTKYGPVKGDSIVEKEEIPFEKERKFNPDL<br>APGTEKVTREGQKGEKTITTPTLKNPLTGEIISKGESKEEITKDPINELTEYGPETIAPGHRDEFDPKLPTGEKEEVPGKPGIKNPETGDVVRP<br>PVDSVTKYGPVKGDSIVEKEEIPFEKERKFNPDLAPGTEKVTREGQKGEKTITTPTLKNPLTGEIISKGESKEEITKDPINELTEYGPETIAPG

	HRDEFDPKLPTGEKEEVPGKPGIKNPETGDVVRPPVDSVTKYGPVKGDSIVEKEEIPFKKERKFNPDLAPGTEKVTREGQKGEKTITTPTLKNP<br>LTGEIISKGESKEEITKDPINELTEYGPETITPGHRDEFDPKLPTGEKEEVPGKPGIKNPETGDVVRPPVDSVTKYGPVKGDSIVEKEEIPFEK<br>ERKFNPDLAPGTEKVTREGQKGEKTITTPTLKNPLTGEIISKGESKEEITKDPVNELTEFGGEKIPQGHKDIFDPNLPTDQTEKVPGKPGIKNP<br>DTGKVIEEPVDDVIKHGPKTGTPETKTVEIPFETKREFNPKLQPGEERVKQEGQPGSKTITTPITVNPLTGEKVGEGQPTEEITKQPVDKIVEF<br>GGEKPKDPKGPENPEKPSRPTHPSGPVNPNNPGLSKDRAKPNGPVHSMDKNDKVKKSKIAKESVANQEKKRAELPKTGLESTQKGLIFSSIIGI<br>AGLMLLARRRKN
**10**	**Q7A6P2, Thermonuclease**<br>MTEYLLSAGICMAIVSILLIGMA↓<br>ISNVSKGQYAKRFFFFATSCLVLTLVVVSSLSSSANA↓**SQTDNGVNR**SGSEDPTVYSATSTKKLHKEPATLIKAIDGDTVKLMYKGQPMTF<br>RLLLVDTPETKHPKKGVEKYGPEASAFTKKMVENANKIEVEFDKGQRTDKYGRGLAYIYADGKMVNEALVRQGLAKVAYVYKPNNTHEQLLRKS<br>EAQAKKEKLNIWSEDNADSGQ
**11**	**A0A0H3JNR9, Uncharacterized protein**<br>MRENFKLRKMKVGLVSVAITMLYIMTNGQAEA↓**SETNQKVSTNQESK**AVSQTEQNSKETKAAESSKNFVNLDPIKPGAQKVTGTTLPNHIILL<br>NIDGKSADSVEGGYGDFITANDKGEFEYPLNNRKIVHNQEIEVSSSSPDLGDDEEDEEVEESSTEKAGAEEESTDAKATYTTPRYEKAYEIPKE<br>QLKEKDGHHQVFIEPITEGSGIIKGHTSVKGKVAL↓**SINNKFINFEER**AKGGISKEDTKASSDGVWMPINEKGYFDFDFKKNPFDNLELKKND<br>EISLTFAPDDEDEALKSLIFKTKVTSLEDIDKAETKYDHTKVEKVKVLKDVKEDIHVDEIYGSLYHTEKGKGILDKQGTKEITGKTKFANAVVK<br>VYSDLGDAQLFPDIQVDENGKFSFDAEKAGFRLQNGETLNFAVVKPITGDLLHQGFVSKYIDVYESPEEKKEREFEEKLENTPAYHKLHGDKIV<br>GYDVQGNPSTWFYPLGEKKVERTTPKLEK
**12**	**A0A0H3JKR2, Penicillin binding protein 2 prime**<br>MKKIKIVPLILIVVVVGFGIYFYASKDKEINNTIDAIEDKNFKQVYKDSSYISKSDNGEVEMTERPIKIYNSLGVKDINIQDRKIKKVSKNKKR<br>VDAQYKIKTNYGNIDRNVQFNFVKEDGMWKLDWDHSVIIPGMQKDQSIHIENLKSERGKILDRNNVELANTGTAYEIGIVPKNVSKKDYKAIAK<br>ELSISEDYIKQQMDQNWVQDDTFVPLKTVKKMDEYLSDFAKKFHLTTNETESRNYPLGKATSHLLGYVGPINSEELKQKEYKGYKDDAVIGKKG<br>L↓EKLYDKKLQHEDGYRVTIVDDNSNTIAHTLIEKKKKDGKDIQLTIDAKVQKSIYNNMKNDYGSGTAIHPQTGELLALVSTPSYDVYPFMYG<br>MSNEEYNKLTEDKKEPLLNKFQITTSPGSTQKILTAMIGLNNKTLDDKTSYKIDGKGWQKDKSWGGYNVTRYEVVNGNIDLKQAIESSDNIFFA<br>RVALELGSKKFEKGMKKLGVGEDIPSDYPFYNAQISNKNLDNEILLADSGYGQGEILINPVQILSIYSALENNGNINAPHLLKDTKNKVWKKNI<br>ISKENINLLTDGMQQV↓**VNKTHKEDIYR**SYANLIGKSGTAE↓**LKMKQGETGR**QIGWFISYDKDNPNMMMAINVKDVQDKGMASYNAKISGKV<br>YDELYENGNKKYDIDE
**13**	**A0A0H3JTB6, Uncharacterized protein**<br>MTVKNLFLGFVAVILTVCLIGLLILATNEDALAKVHKTINTLNAINVSTEDTYKKKMDILNIHTAKA↓SEVNENVEKQNHFKHRVNANKSNSF<br>NEQECQVIADRYADKHINDNYGLERISKTNHGYNYVYSNDNSTSKQHVSISNQGIITK
**14**	**A0A0H3JNE5, Putative long chain fatty acid-CoA ligase VraA**<br>MIMGNLRFQQEYFRIYKNNTESTTHRNAYWVKLAKNVEATKMMYALSTIVQQHASIRHFFDVTTDDNLTMILHEFLPFIEIKQVPSSSANYDLE<br>AFFKQELSTYHFNDSPLFKVKLFQFADAAYILLDFHVSIFDDSQIDIFLDDLCNAYRGNTVINNTRQHAHINRNDDKDNQDASHIALDSNYFRL

	ENNSDIHIDSYFPIKHPFEQALYQTYLIDDMTSIDMASLAVSVYLANHIMSQQHDVTLGIHVPSHLPNDLHGNIVPLTLTIDAKDVCQRFTTDF<br>NKCVLQNMSQLQCAKSSLSLETIFHCYHHMMSCCNDVIEDVHQIHDAHTSLADIEIFPHQHGFKIIYNSAAYDLLSIETLSDLVRNIYLQITEE<br>NGNKRTTVDELNLMTERDIQLYDDINLSLPEIDDAQTVVTLFEQQVEATPNHVAVQFDGVFITYQTLNARANDLAHRLRNQYGVEPNDRVAVIA<br>EKSIEMIIAMIGVLKAGGAYVPIDPNYPSDRQEYILKDATPKVVITYQALYENGKQNINHIDLNKIAWKNIDNLSKCNTLEDHAYVIYTSGTTG<br>NPKGTLIPHRGIVRLVHRNHYVPLNEETTILLSGTIAFDAATFEIYGALLNGGKLIVAKKEQLLNPIAVEQLINENDVNTMWLTSSLFNQIASE<br>RIEVLVPLKYLLIGGEVLNAKWVDLLNQKPKHPQIINGYGPTENTTFTTTYNIPNKVPNRIPIGKPILGTHVYIMQGERRCGVGIPGELCTSGF<br>GLAAGYLNQPELTADKFIKDSNINQLMYRSGDIVRLLPDGNIDYLYRKDKQVKIRGFRIELSEVEHALERIQGINKAVVIVQNHDQDQYIVAYY<br>EAMHTLSHNKIKSQLRMTLPEYMIPVNFMHIEQIPITINGKLDKKALPIMDYVDTDAYVAPSTDTEHLLCQIFADILHVNQVGIHDNFFELGGH<br>SLKATLVVNRIEASTGKRLQIGDLLQKPTVFELAQAIAKVQEQNYEVIPEAIVKDDYVLSSAQKRMYLLWKSNHKDTVYNVPFLWRLSSGLNVA<br>QLQQAVQHLIARHEILRTQYIVVDDEVRQRIVADVVADFEEVNTHFTDEQEIMRQFVAPFNLEKPSQIRVRYIRSPLHAYLFIDTHHIINDGMS<br>NIQLMNDLNALYQHKLLLPLKLQYKDYSEWMSHRDMTKHRQYWLSQFKDEVPILSLPTDYVRPNIKTTNGAMMSFTMNQQMRQLLQKYVEKHQI<br>TDFMFFMSVVMTLLSRYARKDDVVVGSVMSARMHKGTEQMLGMF↓**ANTLVYR**GQPSPDKMWTQFLQEVKEMSLEAYEHQEYPFECLVNDLDQS<br>HDASRNPLFDVMLVLQNNETNHAHFGHSKLTHIQPKSVTAKFDLSFIIEEDRDDYTINIEYNTDLYHSETVRHMGNQCMIMIDYILKHQDTLQI<br>CDIPNGTEELLNWVNTHVNDRMLNVPGNKSIISYFNEVVSRQGNHVALVMNDLTMTYETLRNYVDAIAHMLLSNGVGNGQRVALFTERSFEMIA<br>AMLATVKVGASYIPIDIDFPNKRQGAILEDAKVTAVMSYGVEIETTLPVIQLENAKGFVESKENEQYDDLHGNQLENTAMLDNEMYAIYTSGTT<br>GMPKGVAIRQRNLLNLVHAWSTELQLGDNEVFLQHANIVFDASVMEIYCCLLNGHTLVIPDREERVNPEQLQQLINKHRVTVASIPLQMCSIME<br>DFYIEKLITGGATSTASFVKYIEKHCGTYFNAYGPSESTVITSYWSHHCGDLIPETIPIGKPLSNIQVYIMSDGLLCGIGMPGELCIAGDSLAI<br>GYINRPELMADKWQNNPFGKGKLYHSGDLARYTSDGQIEFLGRIDKQVKVNGYRIELDEIENVILAIRGISDCVVTVSHFDTHDILNAYYVGEQ<br>QVEQDLKQYLNDQLPKYMIPKTITHIDCMPLTTNDKVDTTRLPNPSPIQQSNKVYSEPSNEIEQTFVDVFGEVLKQNDVGVDDDFFELGGNSLE<br>AMLVVSHLKRFGHHISMQTLYQYKTVRQIVNYMYQNQQSLVALPDNLSELQKIVMSRYNLGILEDSLSHRPLGNTLLTGATGFLGAYLIEALQG<br>YSHRIYCFIRADNEEIAWYKLMTNLNDYFSEETVEMMLSNIEVIVGDFECMDDVVLPENMDTIIHAGARTDHFGDDDEFEKVNVQGTVDVIRLA<br>QQHHARLIYVSTISVGTYFDIDTEDVTFSEADVYKGQLLTSPYTRSKFYSELKVLEAVNNGLDGRIVRVGNLTSPYNGRWHMRNIKTNRFSMVM<br>NDLLQLDCIGVSMAEMPVDFSFVDTTARQIVALAQVNTPQIIYHVLSPNKMPVKSLLECVKRKEIELVSDESFNEILQKQDMYETIGLTSVDRE<br>QQLAMIDTTLTLKIMNHISEKWPTITNNWLYHWAQYIKTIFNK
**15**	**P64416, Histidine ammonia-lyase**<br>MTLYLDGETLTIEDIKSFLQQQSKIEIIDDALERVKKSRAVVERIIENEETVYGITTGFGLFSDVRIDPTQYNE↓**LQVNLIR**SHACGLGEPFS<br>KEVALVMMILRLNTLLKGHSGATLELVRQLQFFINERIIPIIPQQGSLGASGDLAPLSHLALALIGEGKVLYRGEEKDSDDVLRELNRQPLNLQ<br>AKEGLALINGTQAMTAQGVISYIEAEDLGYQSEWIAALTHQSLNGIIDAYRHDVHSVRNFQEQINVAARMRDWLEGSTLTTRQAEIRVQDAYTL<br>RCIPQIHGASFQVFNYVKQQLEFEMNAANDNPLIFEEANETFVISGGNFHGQPIAFALDHLKLGVSELANVSERRLERLVNPQLNGDLPAFLSP<br>EPGLQSGAMIMQYAAASLVSENKTLAHPASVDSITSSANQEDHVSMGTTAARHGYQIIENARRVLAIECVIALQAAELKGVEGLSPKTRRKYEE<br>FRSIVPSITHDRQFHKDIEAVAQYLKQSIYQTTACH
**16**	**A0A0H3JQ77, Penicillin-binding protein 3**<br>MLKRLKEKSNDEIVQNTINKRINFIFGVIVFIFAVLVLRLGYLQIAQGSHYKQIIKNDENITVNESVPRGRILDRNGKVLVDNASKMAITYTRG<br>RKTTQSEMLDTAEKLSKLIKMDTKKITERDKKDFWIQLHPKKAKAMMTKEQAMLADGSIKQDQYDKQLLSKIGKSQLDELSSKDLQVLAIFREM<br>NAGTVLDPQMIKNEDVSEKEYAAVSQQLSKLPGVNTSMDWDRKYPYGDTLRGIFGDVSTPAEGIPKELTEHYLSKGYSRNDRVGKSYLEYQYED<br>VLRGKKKEMKYTTDKSGKVTSSEVLNPGARGQDLKLTIDIDLQKEVEALLDKQIKKLRSQGAKDMDNAMMVVQNPKNGDILALAGKQINKSGKM<br>TDYDIGTFTSQFAVGSSVKGGTLLAGYQNKAIKV↓**GETMVDEPLHFQGGLTKR**SYFNKNGHVSINDKQALMHSSNVYMFKTALKLAGDPYYSG<br>MALPSDISSPAQKLRRGLNQVGLGVKTGIDLPNETRGQIEPLTNNPGNYLDLSIGQYDTYTPLQLSQYVSTIANDGYRIQPHIGLTIHESTNKD

	EVGPLKKKINGTVLNKVNNTEKEIKQIQEGFKMAFNDKDGTGYVSFKDTVVPTAGKTGTAEVFQNGEPRVNSTYIGYAPIDDPKLAFSIVYTNQ<br>PVPPPWLTGGDLGRDVINYYFKQLGKDDKNKDKDK
**17**	**A0A0H3JKY5, SA1224 protein**<br>MLQVTDVSLRFGDRKLFEDVNIKFTEGNCYGLIGANGAGKSTF↓**LKILSGELDSQTGHVSLGKNER**LAVLKQDHYAYEDERVLDVVIKGHERL<br>YEVMKEKDEIYMKPDFSDEDGIRAAELEGEFAEMNGWNAEADAANLLSGLGIDPTLHDKKMAELENNQKIKVLLAQSLFGEPDVLLLDEPTNGL<br>DIPAISWLEDFLINFDNTVIVVSHDRHFLNNVCTHIADLDFGKIKVYVGNYDFWYQSSQLAQKMAQEQNKKKEEKMKELQDFIARFSANASKSK<br>QATSRKKQLEKIELDDIQPSSRRYPFVKFTPEREIGNDLLIVQNLSKTIDGEKVLDNVSFTMNPNDKAILIGDSEIAKTTLLKILAGEMEPDEG<br>SFKWGVTTSLSYFPKDNSEFFEGVNMNLVDWLRQYAPEDEQTETFLRGFLGRMLFSGEEVKKKASVLSGGEKVRCMLSKMMLSSANVLLLDEPT<br>NHLDLESITAVNDGLKSFKGSIIFTSYDFEFINTIANRVIDLNKQGGVSKEIPYEEYLQEIGVLK
**18**	**Q7A600, Probable dual-specificity RNA methyltransferase RlmN**<br>MITAEKKKKNKFLPNFDKQSIYSLRFDEMQNWLVEQGQQKFRAKQIFEWLYQKRVDSIDEMTNLSKDLRQLLKDNFTVTTLTTVVKQESKDGTI<br>KFLFELQDGYTIETVLMRHDYGNSVCVTTQVGCRIGCTFCA↓**STLGGLK**RNLEAGEIVSQVLTVQKALDATEERVSQIVIMGIGEPFENYDEM<br>MDFLRIVNDDNSLNIGARHITVSTSGIIPRIYDFADEDIQINFAVSLHAAKDEVRSRLMPINRAYNVEKLIEAIQYYQEKTNRRVTFEYGLFGG<br>VNDQLEHARELAHLIKGLNCHVNLIPVNHVPERNYVKTAKNDIFKFEKELKRLGINATIRREQGSDIDAACGQLRAKERQVETR
**19**	**P99135, Phosphoglycerate kinase**<br>MAKKIVSDLDLKGKTVLVRADFNVPLKDGEITNDNRIVQALPTIQYIIEQGGKIVLFSHLGKVKEESDKAKLTLRPVAEDLSKKLDKEVVFVPE<br>TRGEKLEAA↓**IKDLKEGDVLLVENTR**YEDLDGKKESKNDPELGKYWASLGDVFVNDAFGTAHREHASNVGISTHLETAAGFLMDKEIKFIGGV<br>VNDPHKPVVAILGGAKVSDKINVIKNLVNIADKIIIGGGMAYTFLKAQGKEIGISLLEEDKIDFAKDLLEKHGDKIVLPVDTKVAKEFSNDAKI<br>TVVPSDSIPADQEGMDIGPNTVKLFADELEGAHTVVWNGPMGVFEFSNFAQGTIGVCKAIANLKDAITIIGGGDSAAAAISLGFENDFTHISTG<br>GGASLEYLEGKELPGIKAINNK
**21**	**A0A0H3JM99, Uncharacterized protein**<br>MSILT↓**IILIALLVILLFR**VGLSILRFLIYVGLVLLCIYLGYQGLIWLLDFFQINSGFLPHFQFN
**22**	**P99108, Cell division protein FtsZ**<br>MLEFEQGFNHLATLKVIGVGGGGNNAVNRMIDHGMNNVEFIAINTDGQALNLSKAESKIQIGEKLTRGLGAGANPEIGKKAAEESREQIEDAIQ<br>GADMVFVTSGMGGGTGTGAAPVVAKIAKEMGALTVGVVTRPFSFEGRKRQTQAAAGVEAMKAAVDTLIVIPNDRLLDIVDKSTPMMEAFKEADN<br>VLRQGVQGISDLIAVSGEVNLDFADVKTIMSNQGSALMGIGVSSGENRAVEAAKKAISSPLLETSIVGAQGVLMNITGGESLSLFEAQEAADIV<br>QDAADEDVNMIFGTVINPELQDEIVVTVIATGFDDKPTSHGRKSGSTGFGTSVNTSSNATSKDESFT↓**SNSSNAQATDSVSER**THTTKEDDIP<br>SFIRNREERRSRRTRR
**23**	**A0A0H3JNV0, SA2202 protein**<br>MKRLLFVMIAFVFILAACGNNSSK↓**DKEANKDSK**TINVGTEGTYAPFSFHDKDGKLTGYDIDVIKAVAKEEGLKLKFNETSWDSMFAGLDAGR<br>FDVIANQVGINPDREKKYKFSKPYTFSSAVLVIRENEKDIKDFDDVKGKKLAQTFTSNYGKLAKDKGADITKVDGFNQSMDLLLSKRVDGTFND<br>SLSYLDYKKQKPN↓**AKIKAIKGNAEQSR**SAFAFSKKADDETVQKFNDGLKKIEENGELAKIGKKWFGQDVSKSK

**24**	**A0A0H3JMK9, SA1273 protein**<br>MPNKILLVDGMALLFRHFYATSLHKQFMYNSQGVPTNGIQGFVRHIFSAIHEIRPTHVAVCWDMGQSTFRNDMFDGYKQNRSAPPEELIPQFDY<br>VKEISEQFGFVNIGVKNYEADDVIGTLAQQYSTDNDVYIITGDKDLLQCINDNVEVWLIKKGFNIYNRYTLHRFNEEYALEPQQLIDIKAFMGD<br>TADGYAGVKGIGEKTAIKLIQQYQSVENVVENID↓**ALSAGQR**NKINDNLDELYLSKRLAEIHTQVPIDSEALFEKMSFATTLNHILSICNEHE<br>LHVSGKYISSHF
**25**	**Q7A423, Staphylococcal secretory antigen ssaA2**<br>MKKIATATIATAGFATIAIASGNQAHA↓SEQDNYGYNPNDPTSYSYTYTIDAQGNYHYTWKGNWHPSQLNQDNGYYSYYYYNGYNNYNNYNNG<br>YSYNNYSRYNNYSNNNQSYNYNNYNSYNTNSYRTGGLGASYSTSSNNVQVTTTMAPSSNGRSISSGYTSGRNLYTSGQCTYYVFDRVGGKIGST<br>WGNASNWANAAARAGYTVNNTPKAGAIMQTTQGAYGHVAYVESVNSNGSVRVSEMNYGY↓**GPGVVTSR**TISASQAAGYNFIH
**26**	**A0A0H3JK15, Uncharacterized protein**<br>MKKLLTASIIACSVVMGVGLVNTSAEA↓ASGNSIDTVKQLIKGDQSLENVKIGESIKDVLTKYKNPMYSYNEDGTEHYYEFHTKKGMLLVTTD<br>GKKNNGKVTHISMMYNDANGPTYQAVKNYVGKAVTHTEY↓**SKVAGNFGYIEKGK**TTYQFASAPKDKNIKLYRIDLEK
**27**	**A0A0H3JM43, TPR_REGION domain-containing protein**<br>MNIDKQFWKTIYYWIRYLNFDIVSREKDDQEIWLAHKRKKQVVIFKQHIKSTQEIRFDKAKVLEHKDEIANFISFEPQSFEFYYFTESEFSEEQ<br>LNEVSPIRIKFNVIRHTKDLIKHMPNIFLARLISEDNDKKTYMFYKRKVLTDNFLDKYMQKFSPATYTIIFVNVLIWLCMILYLNNFSDVKLLD<br>VGGLVHFNVVHGEWYRIVTSMFLHFSFEHILMNMLSLFIFGKIVEAIIGSWRMLTVYFIAGLFGNFVSLSFNTTTISVGASGAIFGLIGSIFAM<br>MYVSKTFNKKMLGQLLIALVILVGVSLFMSNINIVAHIGGFIGGLLITLIGYYYKVNRNIFWILLIGMLVIFIALQIRIFTIKEDNIYNKLIKD<br>DMTSGNYDNAQNIVKQTINKNYADDQTYYLS↓**GMIMATINSKSEGMTEWER**GLRMFPKSGLLNFELAIANRSLNDDEKALKYVRKALNADPKN<br>TDYINLEKELTKSNESKNK
**28**	**A0A0H3JTW9, Cell division protein FtsL**<br>MAVEKVYQPYDEQVYNSIPKQQPQTKPEKKTVSRKVVVQLTKFEKVLYITLITVIAMLSIYMLSLKMDAYDTRGKIADLDYKIDKQSSENSALQ<br>SEIKKNSSYERIYEKAKKQGM↓**SLENDNVKVVR**SNGEAKN
**29**	**A0A0H3JPQ1, SA1000 Fibrinogen-binding protein**<br>MKKNFIGKSILSIAAISLTVSTFAGESHA↓**QTKNVEAAKK**YDQYQTNFKKQVNKKVVDAQKAVNLFKRTRTVATHRKAQRAVNLIHFQHSYEK<br>KKLQRQIDLV↓**LKYNTLK**
**30**	**P60432, 50S ribosomal protein L2**<br>MAIKKYKPITNGRRNMTSLDFAEITKTTPEKSLLKPLPKKAGRNNQGKLTVRHHGGGHKRQYRVIDFKRNKDGINAKVDSIQYDPNRSANIALV<br>VYADGEKRYIIAPKGLEVGQIVESGAEADIKVGNALPLQNIPVGTVVHNIELKPGKGGQIARSAGASAQVLGKEGKYVLIRLRSGEVRMILSTC<br>RATIGQVGNLQHELVNVGKAGRSRWKGIRPTVRG↓**SVMNPNDHPHGGGEGR**APIGRPSPMSPWGKPTLGKKTRRGKKSSDKLIVRGRKKK

**31**	**P99152, Elongation factor Tu**<br>MAKEKFDRSKEHANIGTIGHVDHGKTTLTAAIATVLAKNGDSVAQSYDMIDNAPEEKERGITINTSHIEYQTDKRHYAHVDCPGHADYVKNMIT<br>GAAQMDGGILVVSAADGPMPQTREHILLSRNVGVPALVVFLNKVDMVDDEELLELVEMEVRDLLSEYDFPGDDVPVIAGSALKALEGDAQYEEK<br>ILELMEAVDTYIPTPERDSDKPFMMPVEDVFSITGRGTVATGRVERGQIKVGEEVEIIGLHDTSKTTVTGVEMFRKLLDYAEAGDNIGALLRGV<br>AREDVQRGQVLA↓**APGSITPHTEFKAEVYVLSKDEGGR**HTPFFSNYRPQFYFRTTDVTGVVHLPEGTEMVMPGDNVEMTVELIAPIAIEDGTR<br>FSIREGGRTVGSGVVTEIIK
**32**	**P60185, Adapter protein MecA**<br>MRIERVDDTTVKLFITYSDIEARGFSREDLWTNRKRGEEFFWSMMDEINEEEDFVVEGPLWIQVHAFEKGVEVTISKSKNEDMMNMSDDDATDQ<br>FDEQVQELLAQTLEGEDQLEELFEQRTKEKEAQGSKRQKSSARKNTRTIIVKFNDLEDVINYAYHSNPITTEFEDLLYMVDGTYYYAVHFDSHV<br>DQEVINDSYSQLLEFAYPTDRTEVYLNDYAKIIMSH↓**NVTAQVRR**YFPETTE

Figure S1

A0A0H3JPQ1 SA1000 Fibrinogen-binding protein

MKKNFIGKSILSIAAISLTVSTFAGESHA29↓30**QTKNVEAAKK**YDQYQTNFKKQVNKKVVDAQK

AVNLFKRTRTVATHRKAQRAVNLIHFQHSYEKKKLQRQIDLV↓103**LKYNTLK**

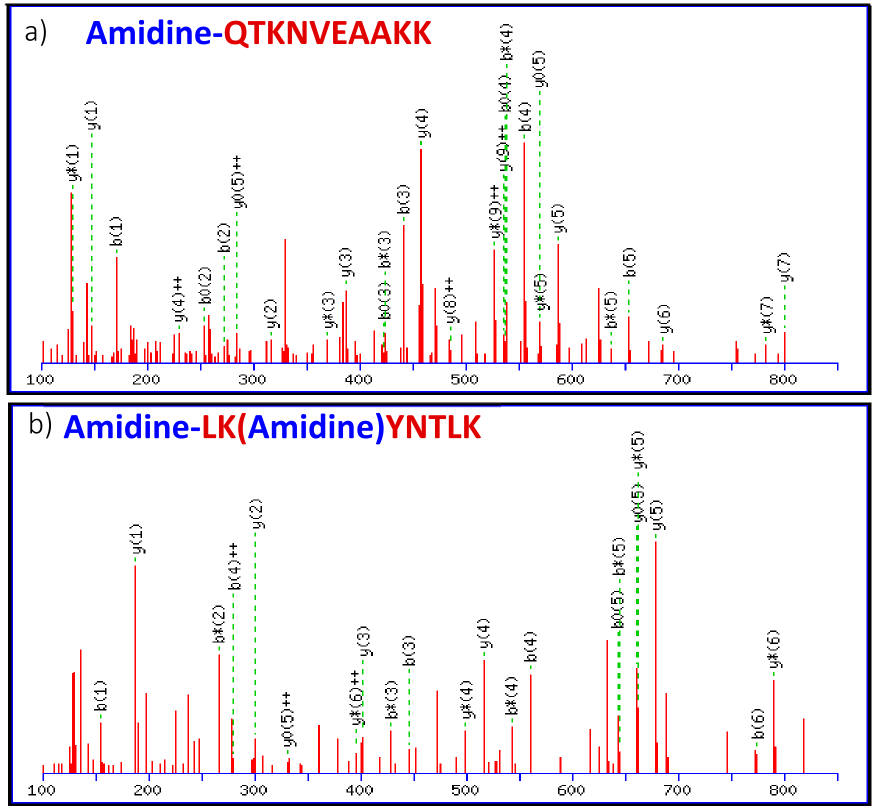

Figure S1. MS/MS spectra of the normal SPase I cleavage and cleavage near to C-terminal. a. MS/MS spectrum of the peptide 30QTKNVEAAKK amidinated at the N-terminus confirms the signal peptide cleavage. b. MS/MS spectrum of the peptide 103LKYNTLK amidinated at the N-terminus and lysine residue. This cleavage occurs near the C-terminus of the protein.

Figure S2.

a) **P99108 Cell division protein FtsZ**

MLEFEQGFNHLATLKVIGVGGGGNNAVNRMIDHGMNNVEFIAINTDGQAL
NLSKAESKIQIGEKLTRGLGAGANPEIGKKAAEESREQIEDAIQGADMVFVTS
GMGGGTGTGAAPVVAKIAKEMGALTVGVVTRPFSFEGRKRQTQAAAGVEA
MKAAVDTLIVIPNDRLLDIVDKSTPMMEAFKEADNVLRQGVQGISDLIAVSG
EVNLDFADVKTIMSNQGSALMGIGVSSGENRAVEAAKKAISSPLLETSIVGAQ
GVLMNITGGESLSLFEAQEAADIVQDAADEDVNMIFGTVINPELQDEIVVTVI
ATGFDDKPTSHGRKSGSTGFGTSVNTSSNATSKDESFT↓**SNSSNAQATDSV**
SERTHTTKEDDIPSFIRNREERRSRRTRR

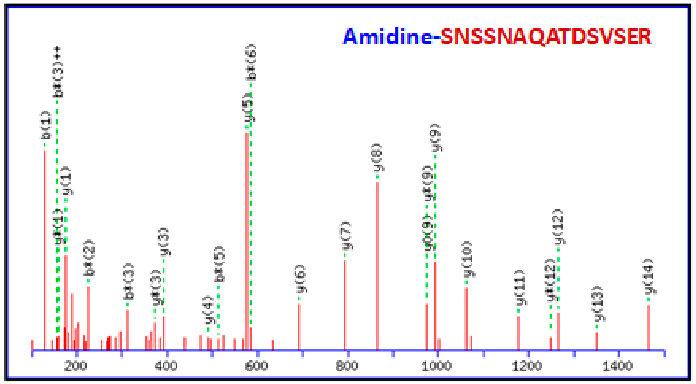

b) **P60432 Ribosomal protein L2**

MAIKKYKPITNGRRNMTSLDFAEITKTTPEKSLLKPLPKKAGRNNQGKLTVR
HHGGGHKRQYRVIDFKRNKDGINAKVDSIQYDPNRSANIALVVYADGEKRYI
IAPKGLEVGQIVESGAEADIKVGNALPLQNIPVGTVVHNIELKPGKGGQIARS
AGASAQVLGKEGKYVLIRLRSGEVRMILSTCRATIGQVGNLQHELVNVGKAG
RSRWKGIRPTVRG↓**SVMNPNDHPHGGGEGR**APIGRPSPMSPWGKPTLG
KKTRRGKKSSDKLIVRGRKKK

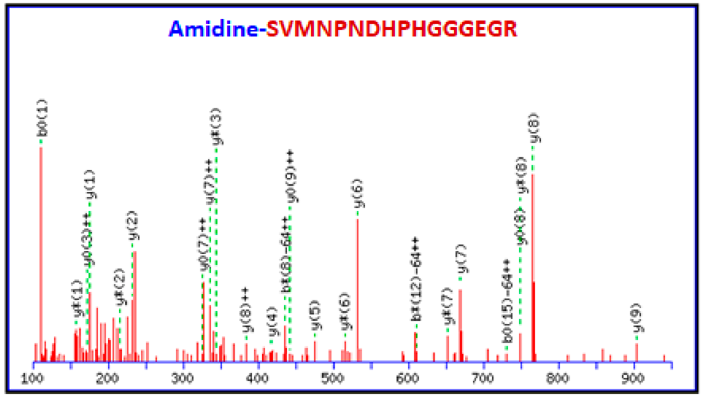

Figure S2. Cytoplasmic proteins are processed middle of their sequence and may be exported outside of the cell

a. MS/MS spectrum of the peptide non-specifically cleaved middle of the proteins. Cell division protein FtsZ is not a secretory protein and does not have the signal peptide. It is present in the inner surface of the cell membrane and is involved in cell division.
b. The MS/MS spectrum of a peptide from ribosomal protein L2. The protein is processed middle of the sequence and exported outside of the cell.

Figure S3. Representative deconvoluted top-down mass spectra

a. **P99134 Immunoglobulin G binding protein A**

MKKKNIYSIRKLGVGIASVTLGTLLISGGVTPAANAAQHDEAQQNAFYQVLNMPNLNADQRNGFIQSLKDDPSQSANVLGE
AQKLNDSQAPKADAQQNNFNKDQQSAFYEILNMPNLNEAQRNGFIQSLKDDPSQSTNVLGEAKKLNESQAPKADNNFNKE
QQNAFYEILNMPNLNEEQRNGFIQSLKDDPSQSANLLSEAKKLNESQAPKADNKFNKEQQNAFYEILHLPNLNEEQRNGFIQS
LKDDPSVSKEILAEAKKLNDAQAPKEEDNKKPGKEDGNKPGKEDGNKPGKEDNKKPGKEDGNKPGKEDNNKPGKEDGNK
PGKEDNNKPGKEDGNKPGKEDGNKPGKEDGNGVHVVKPGDTVNDIAKANGTTADKIAADNKLADKNMIKPGQELVVDKK
QPANHADANKAQALPET↓**GEENPFIGTTVFGGLSLALGAALLAGRRREL**

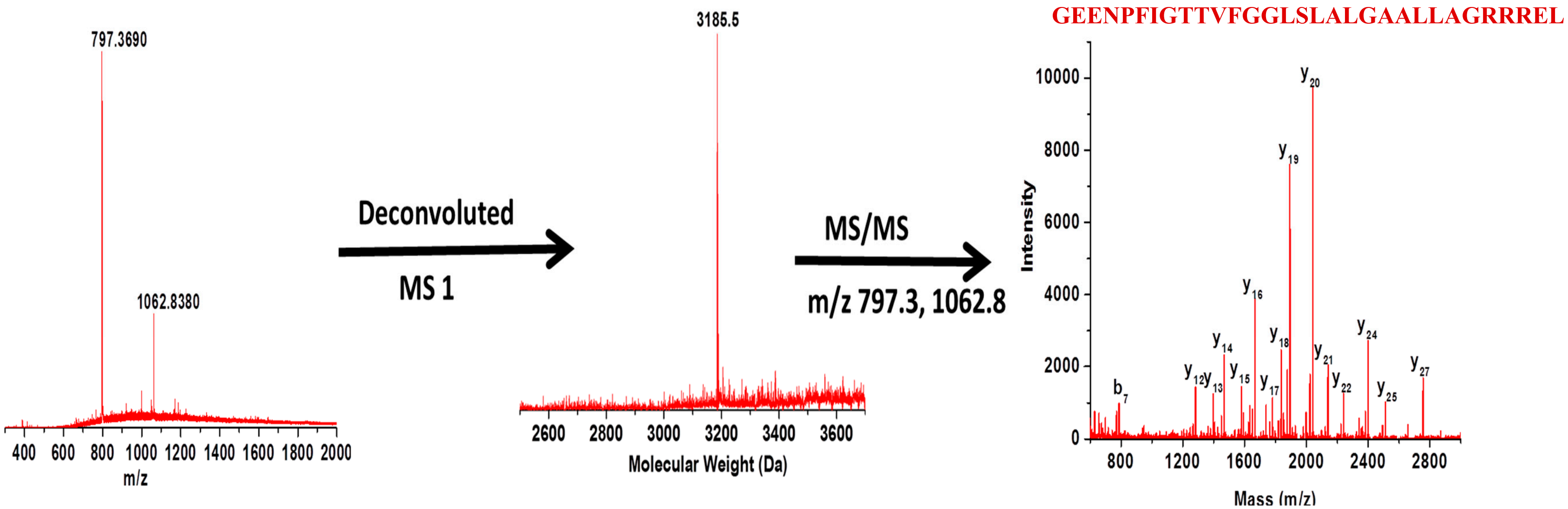

b. Q99SU9 Staphylococcal complement inhibitor

MKIRKSILAGTLAIVLASPLVTNLDKNEAQA↓**STSLPTSNEYQNEKLANELKSLLDELNVNELATGSLNTYYKRTIKISGLKAMYAL KSKDFKKMSEAKYQLQKIYNEIDEALKSKY**

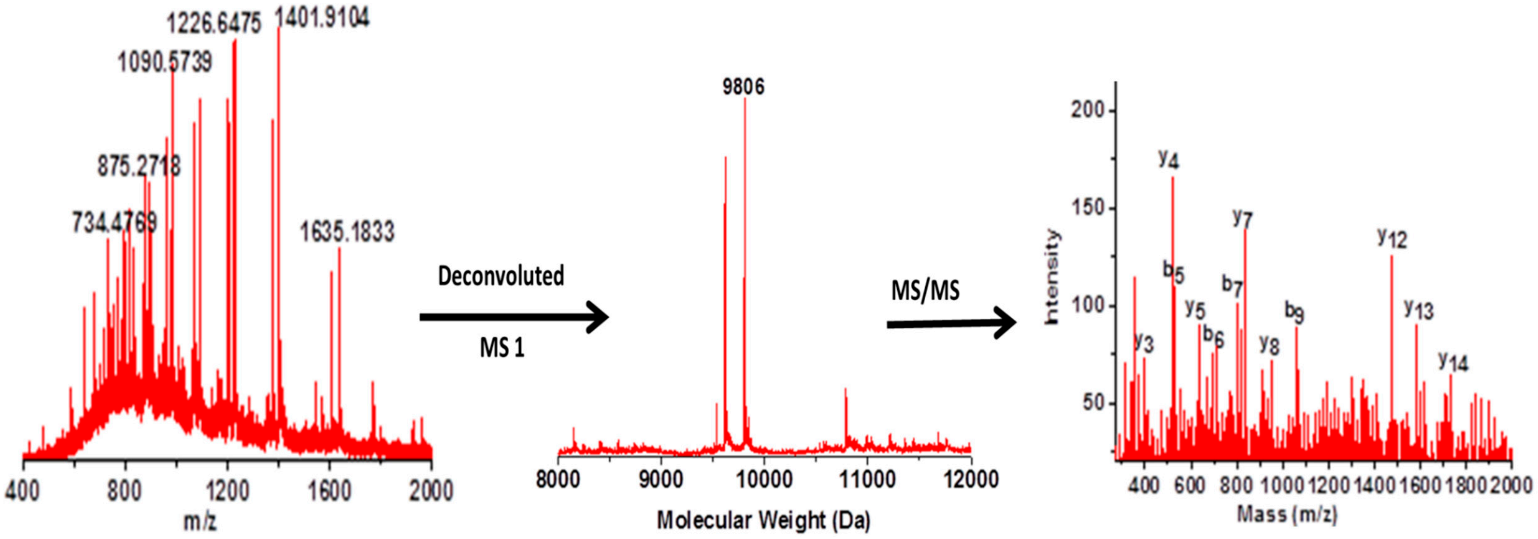

Supplementary Table S4. Proteins and their fragments observed in intact protein analysis. Red colored amino acid sequence is the best match to the observed mass.

Sr. No	Protein Accession, name	Observed Mass (Da)	Protein Sequence
1	P99160, Probable transglycosylase IsaA	9189.4	MKKTIMASSLAVALGVTGYAAGTGHQAHAAEVNVDQAHLVDLAHNHQDQLNAAPIKDGAYD<br>IHFVKDGFQYNFTSNGTTWSWSYEAANGQTAGFSNVAGADYTTSYNQGSDVQSVSYNAQSSN<br>SNVEAVSAPTYHNYSTSTTSSSVRLSNGNTAGATGSSAAQIMAQRTGVSASTWAAIIARESNGQ<br>VNAYNPSGASGLFQTMPGWGPTNTVDQQINAAVKAYKAQGLGAWGF
2		9203(-17Da)	MKKTIMASSLAVALGVTGYAAGTGHQAHAAEVNVDQAHLVDLAHNHQDQLNAAPIKDGAYD<br>IHFVKDGFQYNFTSNGTTWSWSYEAANGQTAGFSNVAGADYTTSYNQGSDVQSVSYNAQSSN<br>SNVEAVSAPTYHNYSTSTTSSSVRLSNGNTAGATGSSAAQIMAQRTGVSASTWAAIIARESNGQ<br>VNAYNPSGASGLFQTMPGWGPTNTVDQQINAAVKAYKAQGLGAWGF
3		10602	MKKTIMASSLAVALGVTGYAAGTGHQAHAAEVNVDQAHLVDLAHNHQDQLNAAPIKDGAYD<br>IHFVKDGFQYNFTSNGTTWSWSYEAANGQTAGFSNVAGADYTTSYNQGSDVQSVSYNAQSSN<br>SNVEAVSAPTYHNYSTSTTSSSVRLSNGNTAGATGSSAAQIMAQRTGVSASTWAAIIARESNGQ<br>VNAYNPSGASGLFQTMPGWGPTNTVDQQINAAVKAYKAQGLGAWGF
4	P99134, Immunoglobulin G binding protein A precursor	3185.5	MKKKNIYSIRKLGVGIASVTLGTLLISGGVTPAANAAQHDEAQQNAFYQVLNMPNLNADQRNG<br>FIQSLKDDPSQSANVLGEAQKLNDSQAPKADAQQNNFNKDQQSAFYEILNMPNLNEAQRNGFI<br>QSLKDDPSQSTNVLGEAKKLNESQAPKADNNFNKEQQNAFYEILNMPNLNEEQRNGFIQSLKD<br>DPSQSANLLSEAKKLNESQAPKADNKFNKEQQNAFYEILHLPNLNEEQRNGFIQSLKDDPSVSK<br>EILAEAKKLNDAQAPKEEDNKKPGKEDGNKPGKEDGNKPGKEDNKKPGKEDGNKPGKEDNN<br>KPGKEDGNKPGKEDNNKPGKEDGNKPGKEDGNKPGKEDGNGVHVVKPGDTVNDIAKANGTT<br>ADKIAADNKLADKNMIKPGQELVVDKKQPANHADANKAQALPETGEENPFIGTTVFGGLSLAL<br>GAALLAGRRREL
5		17579	MKKKNIYSIRKLGVGIASVTLGTLLISGGVTPAANAAQHDEAQQNAFYQVLNMPNLNADQRNG<br>FIQSLKDDPSQSANVLGEAQKLNDSQAPKADAQQNNFNKDQQSAFYEILNMPNLNEAQRNGFI<br>QSLKDDPSQSTNVLGEAKKLNESQAPKADNNFNKEQQNAFYEILNMPNLNEEQRNGFIQSLKD<br>DPSQSANLLSEAKKLNESQAPKADNKFNKEQQNAFYEILHLPNLNEEQRNGFIQSLKDDPSVSK<br>EILAEAKKLNDAQAPKEEDNKKPGKEDGNKPGKEDGNKPGKEDNKKPGKEDGNKPGKEDNN<br>KPGKEDGNKPGKEDNNKPGKEDGNKPGKEDGNKPGKEDGNGVHVVKPGDTVNDIAKANGTT<br>ADKIAADNKLADKNMIKPGQELVVDKKQPANHADANKAQALPETGEENPFIGTTVFGGLSLAL<br>GAALLAGRRREL
6		28807	MKKKNIYSIRKLGVGIASVTLGTLLISGGVTPAANAAQHDEAQQNAFYQVLNMPNLNADQRNG<br>FIQSLKDDPSQSANVLGEAQKLNDSQAPKADAQQNNFNKDQQSAFYEILNMPNLNEAQRNGFI<br>QSLKDDPSQSTNVLGEAKKLNESQAPKADNNFNKEQQNAFYEILNMPNLNEEQRNGFIQSLKD<br>DPSQSANLLSEAKKLNESQAPKADNKFNKEQQNAFYEILHLPNLNEEQRNGFIQSLKDDPSVSK<br>EILAEAKKLNDAQAPKEEDNKKPGKEDGNKPGKEDGNKPGKEDNKKPGKEDGNKPGKEDNN<br>KPGKEDGNKPGKEDNNKPGKEDGNKPGKEDGNKPGKEDGNGVHVVKPGDTVNDIAKANGTT

			ADKIAADNKLADKNMIKPGQELVVDKKQPANHADANKAQALPETGEENPFIGTTVFGGLSLAL GAALLAGRRREL
7		12835	MKKKNIYSIRKLGVGIASVTLGTLLISGGVTPAANAAQHDEAQQNAFYQVLNMPNLNADQRNG FIQSLKDDPSQSANVLGEAQKLNDSQAPKADAQQNNFNKDQQSAFYEILNMPNLNEAQRNGFI QSLKDDPSQSTNVLGEAKKLNESQAPKADNNFNKEQQNAFYEILNMPNLNEEQRNGFIQSLKD DPSQSANLLSEAKKLNESQAPKADNKFNKEQQNAFYEILHLPNLNEEQRNGFIQSLKDDPSVSK EILAEAKKLNDAQAPKEEDNKKPGKEDGNKPGKEDGNKPGKEDNKKPGKEDGNKPGKEDNN KPGKEDGNKPGKEDNNKPGKEDGNKPGKEDGNKPGKEDGNGVHVVKPGDTVNDIAKANGTT ADKIAADNKLADKNMIKPGQELVVDKKQPANHADANKAQALPETGEENPFIGTTVFGGLSLAL GAALLAGRRREL
8	A0A0H3JNG8, staphylocoagulase	13416	MKKQIISLGALAVASSLFTWDNKADAIVTKDYSKESRVNEKSKKGATVSDYYYWKIIDSLEAQF TGAIDLLEDYKYGDPIYKEAKDRLMTRVLGEDQYLLKKKIDEYELYKKWYKSSNKNTNMLTF HKYNLYNLTMNEYNDIFNSLKDAVYQFNKEVKEIEHKNVDLKQFDKDGEDKATKEVYDLVSEI DTLVVTYYADKDYGEHAKELRAKLDLILGDTDNPHKITNERIKKEMIDDLNSIIDDFFMETKQN RPNSITKYDPTKHNFKEKSENKPNFDKLVEETKKAVKEADESWKNKTVKKYEETVTKSPVVKE EKKVEEPQLPKVGNQQEVKTTAGKAEETTQPVAQPLVKIPQETIYGETVKGPEYPTMENKTLQG EIVQGPDFLTMEQNRPSLSDNYTQPTTPNPILEGLEGSSSKLEIKPQGTESTLKGIQGESSDIEVKP QATETTEASQYGPRPQFNKTPKYVKYRDAGTGIREYNDGTFGYEARPRFNKPSETNAYNVTTN QDGTVSYGARPTQNKPSETNAYNVTTHANGQVSYGARPTQKKPSKTNAYNVTTHANGQVSYG ARPTQKKPSKTNAYNVTTHANGQVSYGARPTYKKPSETNAYNVTTHANGQVSYGARPTQKKP SETNAYNVTTHADGTATYGPRVTK
9	P67579, Methionine--tRNA ligase	13176	MAKETFYITTPIYYPSGNLHIGHAYSTVAGDVIARYKRMQGYDVRYLTGTDEHGQKIQEKAQK AGKTEIEYLDEMIAGIKQLWAKLEISNDDFIRTTEERHKHVVEQVFERLLKQGDIYLGEYEGWY SVPDETYYTESQLVDPQYENGKIIGGKSPDSGHEVELVKEESYFFNISKYTDRLLEFYDQNPDFIQ PPSRKNEMINNFIKPGLADLAVSRTSFNWGVHVPSNPKHVVYVWIDALVNYISALGYLSDDESL FNKYWPADIHLMAKEIVRFHSIIWPILLMALDLPLPKKVFAHGWILMKDGKMSKSKGNVVDPNI LIDRYGLDATRYYLMRELPFGSDGVFTPEAFVERTNFDLANDLGNLVNRTISMVNKYFDGELPA YQGPLHELDEEMEAMALETVKSYTESMESLQFSVALSTVWKFISRTNKYIDETTPWVLAKDDS QKDMLGNVMAHLVENIRYAAVLLRPFLTHAPKEIFEQLNINNPQFMEFSSLEQYGVLTESIMVT GQPKPIFPRLDSEAEIAYIKESMQPPATEEEKEEIPSKPQIDIKDFDKVEIKAATIIDAEHVKKSDKL LKIQVDLDSEQRQIVSGIAKFYTPDDIIGKKVAVVTNLKPAKLMGQKSEGMILSAEKDGVLTLVS LPSAIPNGAVIK
10	Q7A7T0, Glycyl-glycine endopeptidase LytM	14901	MKKLTAAAIATMGFATFTMAHQADAAETTNTQQAHTLMSTQSQDVSYGTYYTIDSNGDYHHT PDGNWNQAMFDNKEYSYTFVDAQGHTHYFYNCYPKNANANGSGQTYVNPATAGDNNDYTA SQSQQHINQYGYQSNVGPDASYYSHSNNNQAYNSHDGNGKVNYPNGTSNQNGGSASKATASG HAKDASWLTSRKQLQPYGQYHGGGAHYGVDYAMPENSPVYSLTDGTVVQAGWSNYGGGNQ VTIKEANSNNYQWYMHNNRLTVSAGDKVKAGDQIAYSGSTGNSTAPHVHFQRMSGGIGNQYA VDPTSYLQSR
11		11669	MKKLTAAAIATMGFATFTMAHQADAAETTNTQQAHTLMSTQSQDVSYGTYYTIDSNGDYHHT PDGNWNQAMFDNKEYSYTFVDAQGHTHYFYNCYPKNANANGSGQTYVNPATAGDNNDYTA

			SQSQQHINQYGYQSNVGPDASYYSHSNNNQAYNSHDGNGKVNYPNGTSNQNGGSASKATASG HAKDASWLTSRKQLQPYGQYHGGGAHYGVDYAMPENSPVYSLTDGTVVQAGWSNYGGGNQ VTIKEANSNNYQWYMHNNRLTVSAGDKVKAGDQIAYSGSTGNSTAPHVHFQRMSGGIGNQYA VDPTSYLQSR
12		18495	MKKLTAAAIATMGFATFTMAHQADAAETTNTQQAHTLMSTQSQDVSYGTYYTIDSNGDYHHT PDGNWNQAMFDNKEYSYTFVDAQGHTHYFYNCYPKNANANGSGQTYVNPATAGDNNDYTA SQSQQHINQYGYQSNVGPDASYYSHSNNNQAYNSHDGNGKVNYPNGTSNQNGGSASKATASG HAKDASWLTSRKQLQPYGQYHGGGAHYGVDYAMPENSPVYSLTDGTVVQAGWSNYGGGNQ VTIKEANSNNYQWYMHNNRLTVSAGDKVKAGDQIAYSGSTGNSTAPHVHFQRMSGGIGNQYA VDPTSYLQSR
13	A0A0H3JLB5, SA0295 protein	12910	MNKISKYIAIASLSVAVTVSAPQTTNSTAFAKSSAEVQQTQQASIPASQKANLGNQNIMAVAWY QNSAEAKALYLQGYNSAKTQLDKEIKKNKGKHKLAIALDLDETVLDNSPYQGYASIHNKPFPE GWHEWVQAAKAKPVYGAKEFLKYADKKGVDIYYISDRDKEKDLKATQKNLKQQGIPQAKKS HILLKGKDDKSKESRRQMVQKDHKLVMLFGDNLLDFTDPKEATAESREALIEKHKDDFGKKYII FPNPMYGSWEATIYNNNYKASDKAKDKLRKNAIKQFDPKTGEVK
14		9626	MNKISKYIAIASLSVAVTVSAPQTTNSTAFAKSSAEVQQTQQASIPASQKANLGNQNIMAVAWY QNSAEAKALYLQGYNSAKTQLDKEIKKNKGKHKLAIALDLDETVLDNSPYQGYASIHNKPFPE GWHEWVQAAKAKPVYGAKEFLKYADKKGVDIYYISDRDKEKDLKATQKNLKQQGIPQAKKS HILLKGKDDKSKESRRQMVQKDHKLVMLFGDNLLDFTDPKEATAESREALIEKHKDDFGKKYII FPNPMYGSWEATIYNNNYKASDKAKDKLRKNAIKQFDPKTGEVK
15	A0A0H3JJV0, Exotoxin 15	11595	MKLKNIAKASLALGILTTGMITTTAQPVKASTLEVRSQATQDLSEYYKGRGFELTNVTGYKYG NKVTFIDNSQQIDVTLTGNEKLTVKDDDEVSNVDVFVVREGSDKSAITTSIGGITKTNGTQHKDT VQNVNLSVSKSTGQHTTSVTSEYYSIYKEEISLKELDFKLRKHLIDKHDLYKTEPKDSKIRITMK NGGYYTFELNKKLQPHRMGDTIDSRNIEKIEVNL
16		13003	MKLKNIAKASLALGILTTGMITTTAQPVKASTLEVRSQATQDLSEYYKGRGFELTNVTGYKYG NKVTFIDNSQQIDVTLTGNEKLTVKDDDEVSNVDVFVVREGSDKSAITTSIGGITKTNGTQHKDT VQNVNLSVSKSTGQHTTSVTSEYYSIYKEEISLKELDFKLRKHLIDKHDLYKTEPKDSKIRITMK NGGYYTFELNKKLQPHRMGDTIDSRNIEKIEVNL
17		8516	MKLKNIAKASLALGILTTGMITTTAQPVKASTLEVRSQATQDLSEYYKGRGFELTNVTGYKYG NKVTFIDNSQQIDVTLTGNEKLTVKDDDEVSNVDVFVVREGSDKSAITTSIGGITKTNGTQHKDT VQNVNLSVSKSTGQHTTSVTSEYYSIYKEEISLKELDFKLRKHLIDKHDLYKTEPKDSKIRITMK NGGYYTFELNKKLQPHRMGDTIDSRNIEKIEVNLMKLKNIAKASLALGILTTGMITTTAQPVKAS TLEVRSQATQDLSEYYKGRGFELTNVTGYKYGNKVTFIDNSQQIDVTLTGNEKLTVKDDDEVS NVDVFVVREGSDKSAITTSIGGITKTNGTQHKDTVQNVNLSVSKSTGQHTTSVTSEYYSIYKEEI SLKELDFKLRKHLIDKHDLYKTEPKDSKIRITMKNGGYYTFELNKKLQPHRMGDTIDSRNIEKIE VNL
18	A0A0H3JNR9, Uncharacterized protein	15445	MRENFKLRKMKVGLVSVAITMLYIMTNGQAEASETNQKVSTNQESKAVSQTEQNSKETKAAE SSKNFVNLDPIKPGAQKVTGTTLPNHIILLNIDGKSADSVEGGYGDFITANDKGEFEYPLNNRKIV HNQEIEVSSSSPDLGDDEEDEEVEESSTEKAGAEEESTDAKATYTTPRYEKAYEIPKEQLKEKDG HHQVFIEPITEGSGIIKGHTSVKGKVALSINNKFINFEERAKGGISKEDTKASSDGVWMPINEKGY

			FDFDFKKNPFDNLELKKNDEISLTFAPDDEDEALKSLIFKTKVTSLEDIDKAETKYDHTKVEKVK VLKDVKEDIHVDEIYGSLYHTEKGKGILDKQGTKEITGKTKFANAVVKVYSDLGDAQLFPDIQV DENGKFSFDAEKAGFRLQNGETLNFAVVKPITGDLLHQGFVSKYIDVYESPEEKKEREFEEKLE NTPAYHKLHGDKIVGYDVQGNPSTWFYPLGEKKVERTTPKLEK
19		10282	MRENFKLRKMKVGLVSVAITMLYIMTNGQAEASETNQKVSTNQESKAVSQTEQNSKETKAAE SSKNFVNLDPIKPGAQKVTGTTLPNHIILLNIDGKSADSVEGGYGDFITANDKGEFEYPLNNRKIV HNQEIEVSSSSPDLGDDEEDEEVEESSTEKAGAEEESTDAKATYTTPRYEKAYEIPKEQLKEKDG HHQVFIEPITEGSGIIKGHTSVKGKVALSINNKFINFEERAKGGISKEDTKASSDGVWMPINEKGY FDFDFKKNPFDNLELKKNDEISLTFAPDDEDEALKSLIFKTKVTSLEDIDKAETKYDHTKVEKVK VLKDVKEDIHVDEIYGSLYHTEKGKGILDKQGTKEITGKTKFANAVVKVYSDLGDAQLFPDIQV DENGKFSFDAEKAGFRLQNGETLNFAVVKPITGDLLHQGFVSKYIDVYESPEEKKEREFEEKLE NTPAYHKLHGDKIVGYDVQGNPSTWFYPLGEKKVERTTPKLEK
20		15604	MRENFKLRKMKVGLVSVAITMLYIMTNGQAEASETNQKVSTNQESKAVSQTEQNSKETKAAE SSKNFVNLDPIKPGAQKVTGTTLPNHIILLNIDGKSADSVEGGYGDFITANDKGEFEYPLNNRKIV HNQEIEVSSSSPDLGDDEEDEEVEESSTEKAGAEEESTDAKATYTTPRYEKAYEIPKEQLKEKDG HHQVFIEPITEGSGIIKGHTSVKGKVALSINNKFINFEERAKGGISKEDTKASSDGVWMPINEKGY FDFDFKKNPFDNLELKKNDEISLTFAPDDEDEALKSLIFKTKVTSLEDIDKAETKYDHTKVEKVK VLKDVKEDIHVDEIYGSLYHTEKGKGILDKQGTKEITGKTKFANAVVKVYSDLGDAQLFPDIQV DENGKFSFDAEKAGFRLQNGETLNFAVVKPITGDLLHQGFVSKYIDVYESPEEKKEREFEEKLE NTPAYHKLHGDKIVGYDVQGNPSTWFYPLGEKKVERTTPKLEK
21	A0A0H3JL12, SA0587 protein	12178	MKKLVPLLLALLLLVAACGTGGKQSSDKSNGKLKVVTTNSILYDMAKNVGGDNVDIHSIVPVG QDPHEYEVKPKDIKKLTDADVILYNGLNLETGNGWFEKALEQAGKSLKDKKVIAVSKDVKPIY LNGEEGNKDKQDPHAWLSLDNGIKYVKTIQQTFIDNDKKHKADYEKQGNKYIAQLEKLNNDS KDKFNDIPKEQRAMITSEGAFKYFSKQYGITPGYIWEINTEKQGTPEQMRQAIEFVKKHKLKHLL VETSVDKKAMESLSEETKKDIFGEVYTDSIGKEGTKGDSYYKMMKSNIETVHGSMK
22		13018	MKKLVPLLLALLLLVAACGTGGKQSSDKSNGKLKVVTTNSILYDMAKNVGGDNVDIHSIVPVG QDPHEYEVKPKDIKKLTDADVILYNGLNLETGNGWFEKALEQAGKSLKDKKVIAVSKDVKPIY LNGEEGNKDKQDPHAWLSLDNGIKYVKTIQQTFIDNDKKHKADYEKQGNKYIAQLEKLNNDS KDKFNDIPKEQRAMITSEGAFKYFSKQYGITPGYIWEINTEKQGTPEQMRQAIEFVKKHKLKHLL VETSVDKKAMESLSEETKKDIFGEVYTDSIGKEGTKGDSYYKMMKSNIETVHGSMK
23		12774	MKKLVPLLLALLLLVAACGTGGKQSSDKSNGKLKVVTTNSILYDMAKNVGGDNVDIHSIVPVG QDPHEYEVKPKDIKKLTDADVILYNGLNLETGNGWFEKALEQAGKSLKDKKVIAVSKDVKPIY LNGEEGNKDKQDPHAWLSLDNGIKYVKTIQQTFIDNDKKHKADYEKQGNKYIAQLEKLNNDS KDKFNDIPKEQRAMITSEGAFKYFSKQYGITPGYIWEINTEKQGTPEQMRQAIEFVKKHKLKHLL VETSVDKKAMESLSEETKKDIFGEVYTDSIGKEGTKGDSYYKMMKSNIETVHGSMK
24	A0A0H3JT93, SA0620 protein	12178	MKKLAFAITATSGAAAFLTHHDAQASTQHTVQSGESLWSIAQKYNTSVESIKQNNQLDNNLVF PGQVISVGGSDAQNTSNTSPQAGSASSHTVQAGESLNIIASRYGVSVDQLMAANNLRGYLIMPN QTLQIPNGGSGGTTPTATTGSNGNASSFNHQNLYTAGQCTWYVFDRRAQAGSPISTYWSDAKY WAGNAANDGYQVNNTPSVGSIMQSTPGPYGHVAYVERVNGDGSILISEMNYTYGPYNMNYRT IPASEVSSYAFIH

25	Q7A6A6, Glutamyl endopeptidase	13617	MKGKFLKVSSLFVATLTTATLVSSPAANALSSKAMDNHPQQTQSSKQQTPKIKKGGNLKPLEQ REHANVILPNNDRHQITDTTNGHYAPVTYIQVEAPTGTFIASGVVVGKDTLLTNKHVVDATHG DPHALKAFPSAINQDNYPNGGFTAEQITKYSGEGDLAIVKFSPNEQNKHIGEVVKPATMSNNAE TQVNQNITVTGYPGDKPVATMWESKGKITYLKGEAMQYDLSTTGGNSGSPVFNEKNEVIGIHW GGVPNEFNGAVFINENVRNFLKQNIEDIHFANDDQPNNPDNPDNPNNPDNPNNPDNPNNPDEPN NPDNPNNPDNPDNGDNNNSDNPDAA
26		13002	MKGKFLKVSSLFVATLTTATLVSSPAANALSSKAMDNHPQQTQSSKQQTPKIKKGGNLKPLEQ REHANVILPNNDRHQITDTTNGHYAPVTYIQVEAPTGTFIASGVVVGKDTLLTNKHVVDATHG DPHALKAFPSAINQDNYPNGGFTAEQITKYSGEGDLAIVKFSPNEQNKHIGEVVKPATMSNNAE TQVNQNITVTGYPGDKPVATMWESKGKITYLKGEAMQYDLSTTGGNSGSPVFNEKNEVIGIHW GGVPNEFNGAVFINENVRNFLKQNIEDIHFANDDQPNNPDNPDNPNNPDNPNNPDNPNNPDEPN NPDNPNNPDNPDNGDNNNSDNPDAA
27	Q99V41, Bifunctional autolysin	13002	MAKKFNYKLPSMVALTLVGSAVTAHQVQAAETTQDQTTNKNVLDSNKVKATTEQAKAEVKN PTQNISGTQVYQDPAIVQPKTANNKTGNAQVSQKVDTAQVNGDTRANQSATTNNTQPVAKSTS TTAPKTNTNVTNAGYSLVDDEDDNSEHQINPELIKSAAKPAALETQYKAAAPKAKTEATPKVTT FSASAQPRSVAATPKTSLPKYKPQVNSSINDYIRKNNLKAPKIEEDYTSYFPKYAYRNGVGRPEG IVVHDTANDRSTINGEISYMKNNYQNAFVHAFVDGDRIIETAPTDYLSWGVGAVGNPRFINVEI VHTHDYASFARSMNNYADYAATQLQYYGLKPDSAEYDGNGTVWTHYAVSKYLGGTDHADP HGYLRSHNYSYDQLYDLINEKYLIKMGKVAPWGTQFTTTPTTPSKPTTPSKPSTGKLTVAANNG VAQIKPTNSGLYTTVYDKTGKATNEVQKTFAVSKTATLGNQKFYLVQDYNSGNKFGWVKEGD VVYNTAKSPVNVNQSYSIKSGTKLYTVPWGTSKQVAGSVSGSGNQTFKASKQQQIDKSIYLYG SVNGKSGWVSKAYLVDTAKPTPTPIPKPSTPTTNNKLTVSSLNGVAQINAKNNGLFTTVYDKTG KPTKEVQKTFAVTKEASLGGNKFYLVKDYNSPTLIGWVKQGDVIYNNAKSPVNVMQTYTVKP GTKLYSVPWGTYKQEAGAVSGTGNQTFKATKQQQIDKSIYLFGTVNGKSGWVSKAYLAVPAA PKKAVAQPKTAVKAYTVTKPQTTQTVSKIAQVKPNNTGIRASVYEKTAKNGAKYADRTFYVT KERAHGNETYVLLNNTSHNIPLGWFNVKDLNVQNLGKEVKTTQKYTVNKSNNGLSMVPWGT KNQVILTGNNIAQGTFNATKQVSVGKDVYLYGTINNRTGWVNAKDLTAPTAVKPTTSAAKDY NYTYVIKNGNGYYYVTPNSDTAKYSLKAFNEQPFAVVKEQVINGQTWYYGKLSNGKLAWIKS TDLAKELIKYNQTGMTLNQVAQIQAGLQYKPQVQRVPGKWTDANFNDVKHAMDTKRLAQDP ALKYQFLRLDQPQNISIDKINQFLKGKGVLENQGAAFNKAAQMYGINEVYLISHALLETGNGTS QLAKGADVVNNKVVTNSNTKYHNVFGIAAYDNDPLREGIKYAKQAGWDTVSKAIVGGAKFIG NSYVKAGQNTLYKMRWNPAHPGTHQYATDVDWANINAKIIKGYYDKIGEVGKYFDIPQYK
28		90338	MAKKFNYKLPSMVALTLVGSAVTAHQVQAAETTQDQTTNKNVLDSNKVKATTEQAKAEVKN PTQNISGTQVYQDPAIVQPKTANNKTGNAQVSQKVDTAQVNGDTRANQSATTNNTQPVAKSTS TTAPKTNTNVTNAGYSLVDDEDDNSEHQINPELIKSAAKPAALETQYKAAAPKAKTEATPKVTT FSASAQPRSVAATPKTSLPKYKPQVNSSINDYIRKNNLKAPKIEEDYTSYFPKYAYRNGVGRPEG IVVHDTANDRSTINGEISYMKNNYQNAFVHAFVDGDRIIETAPTDYLSWGVGAVGNPRFINVEI VHTHDYASFARSMNNYADYAATQLQYYGLKPDSAEYDGNGTVWTHYAVSKYLGGTDHADP HGYLRSHNYSYDQLYDLINEKYLIKMGKVAPWGTQFTTTPTTPSKPTTPSKPSTGKLTVAANNG VAQIKPTNSGLYTTVYDKTGKATNEVQKTFAVSKTATLGNQKFYLVQDYNSGNKFGWVKEGD

			VVYNTAKSPVNVNQSYSIKSGTKLYTVPWGTSKQVAGSVSGSGNQTFKASKQQQIDKSIYLYG<br>SVNGKSGWVSKAYLVDTAKPTPTPIPKPSTPTTNNKLTVSSLNGVAQINAKNNGLFTTVYDKTG<br>KPTKEVQKTFAVTKEASLGGNKFYLVKDYNSPTLIGWVKQGDVIYNNAKSPVNVMQTYTVKP<br>GTKLYSVPWGTYKQEAGAVSGTGNQTFKATKQQQIDKSIYLFGTVNGKSGWVSKAYLAVPAA<br>PKKAVAQPKTAVKAYTVTKPQTTQTVSKIAQVKPNNTGIRASVYEKTAKNGAKYADRTFYVT<br>KERAHGNETYVLLNNTSHNIPLGWFNVKDLNVQNLGKEVKTTQKYTVNKSNNGLSMVPWGT<br>KNQVILTGNNIAQGTFNATKQVSVGKDVYLYGTINNRTGWVNAKDLTAPTAVKPTTSAAKDY<br>NYTYVIKNGNGYYYVTPNSDTAKYSLKAFNEQPFAVVKEQVINGQTWYYGKLSNGKLAWIKS<br>TDLAKELIKYNQTGMTLNQVAQIQAGLQYKPQVQRVPGKWTDANFNDVKHAMDTKRLAQDP<br>ALKYQFLRLDQPQNISIDKINQFLKGKGVLENQGAAFNKAAQMYGINEVYLISHALLETGNGTS<br>QLAKGADVVNNKVVTNSNTKYHNVFGIAAYDNDPLREGIKYAKQAGWDTVSKAIVGGAKFIG<br>NSYVKAGQNTLYKMRWNPAHPGTHQYATDVDWANINAKIIKGYYDKIGEVGKYFDIPQYK
29		43355	MAKKFNYKLPSMVALTLVGSAVTAHQVQAAETTQDQTTNKNVLDSNKVKATTEQAKAEVKN<br>PTQNISGTQVYQDPAIVQPKTANNKTGNAQVSQKVDTAQVNGDTRANQSATTNNTQPVAKSTS<br>TTAPKTNTNVTNAGYSLVDDEDDNSEHQINPELIKSAAKPAALETQYKAAAPKAKTEATPKVTT<br>FSASAQPRSVAATPKTSLPKYKPQVNSSINDYIRKNNLKAPKIEEDYTSYFPKYAYRNGVGRPEG<br>IVVHDTANDRSTINGEISYMKNNYQNAFVHAFVDGDRIIETAPTDYLSWGVGAVGNPRFINVEI<br>VHTHDYASFARSMNNYADYAATQLQYYGLKPDSAEYDGNGTVWTHYAVSKYLGGTDHADP<br>HGYLRSHNYSYDQLYDLINEKYLIKMGKVAPWGTQFTTTPTTPSKPTTPSKPSTGKLTVAANNG<br>VAQIKPTNSGLYTTVYDKTGKATNEVQKTFAVSKTATLGNQKFYLVQDYNSGNKFGWVKEGD<br>VVYNTAKSPVNVNQSYSIKSGTKLYTVPWGTSKQVAGSVSGSGNQTFKASKQQQIDKSIYLYG<br>SVNGKSGWVSKAYLVDTAKPTPTPIPKPSTPTTNNKLTVSSLNGVAQINAKNNGLFTTVYDKTG<br>KPTKEVQKTFAVTKEASLGGNKFYLVKDYNSPTLIGWVKQGDVIYNNAKSPVNVMQTYTVKP<br>GTKLYSVPWGTYKQEAGAVSGTGNQTFKATKQQQIDKSIYLFGTVNGKSGWVSKAYLAVPAA<br>PKKAVAQPKTAVKAYTVTKPQTTQTVSKIAQVKPNNTGIRASVYEKTAKNGAKYADRTFYVT<br>KERAHGNETYVLLNNTSHNIPLGWFNVKDLNVQNLGKEVKTTQKYTVNKSNNGLSMVPWGT<br>KNQVILTGNNIAQGTFNATKQVSVGKDVYLYGTINNRTGWVNAKDLTAPTAVKPTTSAAKDY<br>NYTYVIKNGNGYYYVTPNSDTAKYSLKAFNEQPFAVVKEQVINGQTWYYGKLSNGKLAWIKS<br>TDLAKELIKYNQTGMTLNQVAQIQAGLQYKPQVQRVPGKWTDANFNDVKHAMDTKRLAQDP<br>ALKYQFLRLDQPQNISIDKINQFLKGKGVLENQGAAFNKAAQMYGINEVYLISHALLETGNGTS<br>QLAKGADVVNNKVVTNSNTKYHNVFGIAAYDNDPLREGIKYAKQAGWDTVSKAIVGGAKFIG<br>NSYVKAGQNTLYKMRWNPAHPGTHQYATDVDWANINAKIIKGYYDKIGEVGKYFDIPQYK
30		15146	MAKKFNYKLPSMVALTLVGSAVTAHQVQAAETTQDQTTNKNVLDSNKVKATTEQAKAEVKN<br>PTQNISGTQVYQDPAIVQPKTANNKTGNAQVSQKVDTAQVNGDTRANQSATTNNTQPVAKSTS<br>TTAPKTNTNVTNAGYSLVDDEDDNSEHQINPELIKSAAKPAALETQYKAAAPKAKTEATPKVTT<br>FSASAQPRSVAATPKTSLPKYKPQVNSSINDYIRKNNLKAPKIEEDYTSYFPKYAYRNGVGRPEG<br>IVVHDTANDRSTINGEISYMKNNYQNAFVHAFVDGDRIIETAPTDYLSWGVGAVGNPRFINVEI<br>VHTHDYASFARSMNNYADYAATQLQYYGLKPDSAEYDGNGTVWTHYAVSKYLGGTDHADP<br>HGYLRSHNYSYDQLYDLINEKYLIKMGKVAPWGTQFTTTPTTPSKPTTPSKPSTGKLTVAANNG<br>VAQIKPTNSGLYTTVYDKTGKATNEVQKTFAVSKTATLGNQKFYLVQDYNSGNKFGWVKEGD

			VVYNTAKSPVNVNQSYSIKSGTKLYTVPWGTSKQVAGSVSGSGNQTFKASKQQQIDKSIYLYG<br>SVNGKSGWVSKAYLVDTAKPTPTPIPKPSTPTTNNKLTVSSLNGVAQINAKNNGLFTTVYDKTG<br>KPTKEVQKTFAVTKEASLGGNKFYLVKDYNSPTLIGWVKQGDVIYNNAKSPVNVMQTYTVKP<br>GTKLYSVPWGTYKQEAGAVSGTGNQTFKATKQQQIDKSIYLFGTVNGKSGWVSKAYLAVPAA<br>PKKAVAQPKTAVKAYTVTKPQTTQTVSKIAQVKPNNTGIRASVYEKTAKNGAKYADRTFYVT<br>KERAHGNETYVLLNNTSHNIPLGWFNVKDLNVQNLGKEVKTTQKYTVNKSNNGLSMVPWGT<br>KNQVILTGNNIAQGTFNATKQVSVGKDVYLYGTINNRTGWVNAKDLTAPTAVKPTTSAAKDY<br>NYTYVIKNGNGYYYVTPNSDTAKYSLKAFNEQPFAVVKEQVINGQTWYYGKLSNGKLAWIKS<br>TDLAKELIKYNQTGMTLNQVAQIQAGLQYKPQVQRVPGKWTDANFNDVKHAMDTKRLAQDP<br>ALKYQFLRLDQPQNISIDKINQFLKGKGVLENQGAAFNKAAQMYGINEVYLISHALLETGNGTS<br>QLAKGADVVNNKVVTNSNTKYHNVFGIAAYDNDPLREGIKYAKQAGWDTVSKAIVGGAKFIG<br>NSYVKAGQNTLYKMRWNPAHPGTHQYATDVDWANINAKIIKGYYDKIGEVGKYFDIPQYK
31		10789	MAKKFNYKLPSMVALTLVGSAVTAHQVQAAETTQDQTTNKNVLDSNKVKATTEQAKAEVKN<br>PTQNISGTQVYQDPAIVQPKTANNKTGNAQVSQKVDTAQVNGDTRANQSATTNNTQPVAKSTS<br>TTAPKTNTNVTNAGYSLVDDEDDNSEHQINPELIKSAAKPAALETQYKAAAPKAKTEATPKVTT<br>FSASAQPRSVAATPKTSLPKYKPQVNSSINDYIRKNNLKAPKIEEDYTSYFPKYAYRNGVGRPEG<br>IVVHDTANDRSTINGEISYMKNNYQNAFVHAFVDGDRIIETAPTDYLSWGVGAVGNPRFINVEI<br>VHTHDYASFARSMNNYADYAATQLQYYGLKPDSAEYDGNGTVWTHYAVSKYLGGTDHADP<br>HGYLRSHNYSYDQLYDLINEKYLIKMGKVAPWGTQFTTTPTTPSKPTTPSKPSTGKLTVAANNG<br>VAQIKPTNSGLYTTVYDKTGKATNEVQKTFAVSKTATLGNQKFYLVQDYNSGNKFGWVKEGD<br>VVYNTAKSPVNVNQSYSIKSGTKLYTVPWGTSKQVAGSVSGSGNQTFKASKQQQIDKSIYLYG<br>SVNGKSGWVSKAYLVDTAKPTPTPIPKPSTPTTNNKLTVSSLNGVAQINAKNNGLFTTVYDKTG<br>KPTKEVQKTFAVTKEASLGGNKFYLVKDYNSPTLIGWVKQGDVIYNNAKSPVNVMQTYTVKP<br>GTKLYSVPWGTYKQEAGAVSGTGNQTFKATKQQQIDKSIYLFGTVNGKSGWVSKAYLAVPAA<br>PKKAVAQPKTAVKAYTVTKPQTTQTVSKIAQVKPNNTGIRASVYEKTAKNGAKYADRTFYVT<br>KERAHGNETYVLLNNTSHNIPLGWFNVKDLNVQNLGKEVKTTQKYTVNKSNNGLSMVPWGT<br>KNQVILTGNNIAQGTFNATKQVSVGKDVYLYGTINNRTGWVNAKDLTAPTAVKPTTSAAKDY<br>NYTYVIKNGNGYYYVTPNSDTAKYSLKAFNEQPFAVVKEQVINGQTWYYGKLSNGKLAWIKS<br>TDLAKELIKYNQTGMTLNQVAQIQAGLQYKPQVQRVPGKWTDANFNDVKHAMDTKRLAQDP<br>ALKYQFLRLDQPQNISIDKINQFLKGKGVLENQGAAFNKAAQMYGINEVYLISHALLETGNGTS<br>QLAKGADVVNNKVVTNSNTKYHNVFGIAAYDNDPLREGIKYAKQAGWDTVSKAIVGGAKFIG<br>NSYVKAGQNTLYKMRWNPAHPGTHQYATDVDWANINAKIIKGYYDKIGEVGKYFDIPQYK
32	A0A0H3JMD8, SA2094 protein	8962	MMKRKPTFLESISTMIVMVIVVVTGFVFFDIPIQVLLIIASAYATWIAKRVGLTWQDLEKGIAERL<br>NTAMPAILIILAVGIIVGSWMFSGTVPALIYYGLDLLNPSYFLISAFFISAVTS<br>VATGTAWGSASTAGIALISIGNQLGIPPGMAAGAIIAGAVFGDKMSPLSDTTNLAALVTKVNIFK<br>HIHSMMWTTIPASIIGLLVWFIAGFQFKGHSNDKQIQTLLSELAQIYQINIWVWV<br>PLIVIIVCLLFKMATVPAMVISSFSAIIVGTFNHHFKMTDGFKATFSGFNDSMIHQSHISSSVKSLL<br>EQGGMMSMTQILVTIFCGYAFAGIVEKAGCLEVLLTTISKGIHSVGSLICITV<br>ICCIALVFAAGVASIVIIMVGVLMKDLFEKYQVSRSVLSRTLEDSSTMVLPLIPWGTSGIYYTNQL<br>HVSVGEFFMWTVPCYLCAIIAIIYGFTGIGIKKSSNSRLT

33	P68800, Fibrinogen-binding protein	15790	MKNKLIAKSLLTIAAIGITTTTIASTADASEGYGPREKKPVSINHNIVEYNDGTFKYQSRPKFNST PKYIKFKHDYNILEFNDGTFEYGARPQFNKPAAKTDATIKKEQKLIQAQNLVREFEKTHTVSAH RKAQKAVNLVSFEYKVKKMVLQERIDNVLKQGLVR
34		15790	MKNKLIAKSLLTIAAIGITTTTIASTADASEGYGPREKKPVSINHNIVEYNDGTFKYQSRPKFNST PKYIKFKHDYNILEFNDGTFEYGARPQFNKPAAKTDATIKKEQKLIQAQNLVREFEKTHTVSAH RKAQKAVNLVSFEYKVKKMVLQERIDNVLKQGLVR
35		11106	MKNKLIAKSLLTIAAIGITTTTIASTADASEGYGPREKKPVSINHNIVEYNDGTFKYQSRPKFNST PKYIKFKHDYNILEFNDGTFEYGARPQFNKPAAKTDATIKKEQKLIQAQNLVREFEKTHTVSAH RKAQKAVNLVSFEYKVKKMVLQERIDNVLKQGLVR
36	P60748, Foldase protein PrsA	14127	MKMINKLIVPVTASALLLGACGASATDSKENTLISSKAGDVTVADTMKKIGKDQIANASFTEML NKILADKYKNKVNDKKIDEQIEKMQKQYGGKDKFEKALQQQGLTADKYKENLRTAAYHKELL SDKIKISDSEIKEDSKKASHILIKVKSKKSDKEGLDDKEAKQKAEEIQKEVSKDPSKFGEIAKKES MDTGSAKKDGELGYVLKGQTDKDFEKALFKLKDGEVSEVVKSSFGYHIIKADKPTDFNSEKQS LKEKLVDQKVQKNPKLLTDAYKDLLKEYDVDFKDRDIKSVVEDKILNPEKLKQGGAQGGQSG MSQ
37		14901	MKMINKLIVPVTASALLLGACGASATDSKENTLISSKAGDVTVADTMKKIGKDQIANASFTEML NKILADKYKNKVNDKKIDEQIEKMQKQYGGKDKFEKALQQQGLTADKYKENLRTAAYHKELL SDKIKISDSEIKEDSKKASHILIKVKSKKSDKEGLDDKEAKQKAEEIQKEVSKDPSKFGEIAKKES MDTGSAKKDGELGYVLKGQTDKDFEKALFKLKDGEVSEVVKSSFGYHIIKADKPTDFNSEKQS LKEKLVDQKVQKNPKLLTDAYKDLLKEYDVDFKDRDIKSVVEDKILNPEKLKQGGAQGGQSG MSQ
38		14699	MKMINKLIVPVTASALLLGACGASATDSKENTLISSKAGDVTVADTMKKIGKDQIANASFTEML NKILADKYKNKVNDKKIDEQIEKMQKQYGGKDKFEKALQQQGLTADKYKENLRTAAYHKELL SDKIKISDSEIKEDSKKASHILIKVKSKKSDKEGLDDKEAKQKAEEIQKEVSKDPSKFGEIAKKES MDTGSAKKDGELGYVLKGQTDKDFEKALFKLKDGEVSEVVKSSFGYHIIKADKPTDFNSEKQS LKEKLVDQKVQKNPKLLTDAYKDLLKEYDVDFKDRDIKSVVEDKILNPEKLKQGGAQGGQSG MSQ
39		12878	MKMINKLIVPVTASALLLGACGASATDSKENTLISSKAGDVTVADTMKKIGKDQIANASFTEML NKILADKYKNKVNDKKIDEQIEKMQKQYGGKDKFEKALQQQGLTADKYKENLRTAAYHKELL SDKIKISDSEIKEDSKKASHILIKVKSKKSDKEGLDDKEAKQKAEEIQKEVSKDPSKFGEIAKKES MDTGSAKKDGELGYVLKGQTDKDFEKALFKLKDGEVSEVVKSSFGYHIIKADKPTDFNSEKQS LKEKLVDQKVQKNPKLLTDAYKDLLKEYDVDFKDRDIKSVVEDKILNPEKLKQGGAQGGQSG MSQ
40		23875	MKMINKLIVPVTASALLLGACGASATDSKENTLISSKAGDVTVADTMKKIGKDQIANASFTEML NKILADKYKNKVNDKKIDEQIEKMQKQYGGKDKFEKALQQQGLTADKYKENLRTAAYHKELL SDKIKISDSEIKEDSKKASHILIKVKSKKSDKEGLDDKEAKQKAEEIQKEVSKDPSKFGEIAKKES MDTGSAKKDGELGYVLKGQTDKDFEKALFKLKDGEVSEVVKSSFGYHIIKADKPTDFNSEKQS LKEKLVDQKVQKNPKLLTDAYKDLLKEYDVDFKDRDIKSVVEDKILNPEKLKQGGAQGGQSG MSQ

41		2083.2	MKMINKLIVPVTASALLLGACGASATDSKENTLISSKAGDVTVADTMKKIGKDQIANASFTEML NKILADKYKNKVNDKKIDEQIEKMQKQYGGKDKFEKALQQQGLTADKYKENLRTAAYHKELL SDKIKISDSEIKEDSKKASHILIKVKSKKSDKEGLDDKEAKQKAEEIQKEVSKDPSKFGEIAKKES MDTGSAKKDGELGYVLKGQTDKDFEKALFKLKDGEVSEVVKSSFGYHIIKADKPTDFNSEKQS LKEKLVDQKVQKNPKLLTDAYKDLLKEYDVDFKDRDIKSVVEDKI LNPEKLKQGGAQGGQSGMSQ
41	A0A0H3JNJ9, Probable molybdate-binding protein	17366	MKMKRFIAIVMALFLVLAGCSNSNDNNESKKDDADNGKKQEIQVAAAASLTDVTKKLASEFK KEHKNADIKFNYGGSGALRKQIESGAPVDVFMSANTKDVDALKDKNKAHDTYKYAKNSLVLI GDKDSNYTSVKDLKDNDKLALGEVKTVPAGKYAKQYLDNNNLFKEVESKIVYAKDVKQVLN YVEKGNAKQGFVYKTDLYKQNKKIDTVKVIKEVELKKPITYEAGATSDSKLAKEWMEFLKSD KAKEILKEYHFAA
43		19061	MKMKRFIAIVMALFLVLAGCSNSNDNNESKKDDADNGKKQEIQVAAAASLTDVTKKLASEFK KEHKNADIKFNYGGSGALRKQIESGAPVDVFMSANTKDVDALKDKNKAHDTYKYAKNSLVLI GDKDSNYTSVKDLKDNDKLALGEVKTVPAGKYAKQYLDNNNLFKEVESKIVYAKDVKQVLN YVEKGNAKQGFVYKTDLYKQNKKIDTVKVIKEVELKKPITYEAGATSDSKLAKEWMEFLKSD KAKEILKEYHFAA
44		14127	MKMKRFIAIVMALFLVLAGCSNSNDNNESKKDDADNGKKQEIQVAAAASLTDVTKKLASEFK KEHKNADIKFNYGGSGALRKQIESGAPVDVFMSANTKDVDALKDKNKAHDTYKYAKNSLVLI GDKDSNYTSVKDLKDNDKLALGEVKTVPAGKYAKQYLDNNNLFKEVESKIVYAKDVKQVLN YVEKGNAKQGFVYKTDLYKQNKKIDTVKVIKEVELKKPITYEAGATSDSKLAKEWMEFLKSD KAKEILKEYHFAA
45	Q7A423, Staphylococcal secretory antigen ssaA2	12164	MKKIATATIATAGFATIAIASGNQAHASEQDNYGYNPNDPTSYSYTYTIDAQGNYHYTWKGNW HPSQLNQDNGYYSYYYYNGYNNYNNYNNGYSYNNYSRYNNYSNNNQSYNYNNYNSYNTNS YRTGGLGASYSTSSNNVQVTTTMAPSSNGRSISSGYTSGRNLYTSGQCTYYVFDRVGGKIGSTW GNASNWANAAARAGYTVNNTPKAGAIMQTTQGAYGHVAYVESVNSNGSVRVSE MNYGYGPGVVTSRTISASQAAGYNFIH
46		20300	MKKIATATIATAGFATIAIASGNQAHASEQDNYGYNPNDPTSYSYTYTIDAQGNYHYTWKGNW HPSQLNQDNGYYSYYYYNGYNNYNNYNNGYSYNNYSRYNNYSNNNQSYNYNNYNSYNTNS YRTGGLGASYSTSSNNVQVTTTMAPSSNGRSISSGYTSGRNLYTSGQCTYYVFDRVGGKIGSTW GNASNWANAAARAGYTVNNTPKAGAIMQTTQGAYGHVAYVESVNSNGSVRVSEMNYGYGP GVVTSRTISASQAAGYNFIH
47	Q99RL2, Immunoglobulin-binding protein Sbi	11345	MKNKYISKLLVGAATITLATMISNGEAKASENTQQTSTKHQTTQNNYVTDQQKAFYQVLHLKG ITEEQRNQYIKTLREHPERAQEVFSESLKDSKNPDRRVAQQNAFYNVLKNDNLTEQEKNNYIAQ IKENPDRSQQVWVESVQSSKAKERQNIENADKAIKDFQDNKAPHDKSAAYEANSKLPKDLRDK NNRFVEKVSIEKAIVRHDERVKSANDAISKLNEKDSIENRRLAQREVNKAPMDVKEHLQKQLD ALVAQKDAEKKVAPKVEAPQIQSPQIEKPKAESPKVEVPQIQSPKVEVPQSKLLGYYQSLKDSF NYGYKYLTDTYKSYKEKYDTAKYYYNTYYKYKGAIDQTVLTVLGSGSKSYIQPLKVDDKNGY LAKSYAQVRNYVTESINTGKVLYTFYQNPTLVKTAIKAQETASSIKNTLSNLLSFWK

48		16264	MKNKYISKLLVGAATITLATMISNGEAKASENTQQTSTKHQTTQNNYVTDQQKAFYQVLHLKG ITEEQRNQYIKTLREHPERAQEVFSESLKDSKNPDRRVAQQNAFYNVLKNDNLTEQEKNNYIAQ IKENPDRSQQVWVESVQSSKAKERQNIENADKAIKDFQDNKAPHDKSAAYEANSKLPKDLRDK NNRFVEKVSIEKAIVRHDERVKSANDAISKLNEKDSIENRRLAQREVNKAPMDVKEHLQKQLD ALVAQKDAEKKVAPKVEAPQIQSPQIEKPKAESPKVEVPQIQSPKVEVPQSKLLGYYQSLKDSF NYGYKYLTDTYKSYKEKYDTAKYYYNTYYKYKGAIDQTVLTVLGSGSKSYIQPLKVDDKNGY LAKSYAQVRNYVTESINTGKVLYTFYQNPTLVKTAIKAQETASSIKNTLSNLLSFWK
49		11668	MKNKYISKLLVGAATITLATMISNGEAKASENTQQTSTKHQTTQNNYVTDQQKAFYQVLHLKG ITEEQRNQYIKTLREHPERAQEVFSESLKDSKNPDRRVAQQNAFYNVLKNDNLTEQEKNNYIAQ IKENPDRSQQVWVESVQSSKAKERQNIENADKAIKDFQDNKAPHDKSAAYEANSKLPKDLRDK NNRFVEKVSIEKAIVRHDERVKSANDAISKLNEKDSIENRRLAQREVNKAPMDVKEHLQKQLD ALVAQKDAEKKVAPKVEAPQIQSPQIEKPKAESPKVEVPQIQSPKVEVPQSKLLGYYQSLKDSF NYGYKYLTDTYKSYKEKYDTAKYYYNTYYKYKGAIDQTVLTVLGSGSKSYIQPLKVDDKNGY LAKSYAQVRNYVTESINTGKVLYTFYQNPTLVKTAIKAQETASSIKNTLSNLLSFWK
50	P0A075, Gamma-hemolysin component B	10693	MKMNKLVKSSVATSMALLLLSGTANAEGKITPVSVKKVDDKVTLYKTTATADSDKFKISQILTF NFIKDKSYDKDTLVLKATGNINSGFVKPNPNDYDFSKLYWGAKYNVSISSQSNDSVNVVDYAP KNQNEEFQVQNTLGYTFGGDISISNGLSGGLNGNTAFSETINYKQESYRTTLSRNTNYKNVGWG VEAHKIMNNGWGPYGRDSFHPTYGNELFLAGRQSSAYAGQNFIAQHQMPLLSRSNFNPEFLSV LSHRQDGAKKSKITVTYQREMDLYQIRWNGFYWAGANYKNFKTRTFKSTYEIDWENHKVKLL DTKETENNK
51		10300	MKMNKLVKSSVATSMALLLLSGTANAEGKITPVSVKKVDDKVTLYKTTATADSDKFKISQILTF NFIKDKSYDKDTLVLKATGNINSGFVKPNPNDYDFSKLYWGAKYNVSISSQSNDSVNVVDYAP KNQNEEFQVQNTLGYTFGGDISISNGLSGGLNGNTAFSETINYKQESYRTTLSRNTNYKNVGWG VEAHKIMNNGWGPYGRDSFHPTYGNELFLAGRQSSAYAGQNFIAQHQMPLLSRSNFNPEFLSV LSHRQDGAKKSKITVTYQREMDLYQIRWNGFYWAGANYKNFKTRTFKSTYEIDWENHKVKLL DTKETENNK
52		22581	MKMNKLVKSSVATSMALLLLSGTANAEGKITPVSVKKVDDKVTLYKTTATADSDKFKISQILTF NFIKDKSYDKDTLVLKATGNINSGFVKPNPNDYDFSKLYWGAKYNVSISSQSNDSVNVVDYAP KNQNEEFQVQNTLGYTFGGDISISNGLSGGLNGNTAFSETINYKQESYRTTLSRNTNYKNVGWG VEAHKIMNNGWGPYGRDSFHPTYGNELFLAGRQSSAYAGQNFIAQHQMPLLSRSNFNPEFLSV LSHRQDGAKKSKITVTYQREMDLYQIRWNGFYWAGANYKNFKTRTFKSTYEIDWENHKVKLL DTKETENNK
53	P65806, Peptidase T	4763	MKNQLIDRLTRYTTIDTQSDPKSTTTPSTEKQWDLLHLLEKELQQLGLPTDLDENGYLFATLESN IDADVPTVGFLAHVDTSPDFNASNVKPQIIENYDGKPYKLGNTKRVLDPKVFPELNSLVGHTLM VTDGTSLLGADDKAGIVEIMEAICYLQEHPEIKHGTIRIGFTPDEEIGRGPHKFDVDRFNADFAYT MDGSQYGELQYESFNAAEAVITCHGVNVHPGSAKNAMVNAIRLGEQFDSLLPDSEVPERTEGY EGFYHLMNFEGTVEKATLQYIIRDHDKKQFELRKKRILEIRDDINAHFENYPVKVDISDQYFNM AEKILPLPHIIDIPKRVFAKLDIPANTEPIRGGTDGSQLSFMGLPTPNIFTGCGNFHGPYEYASIDV MEKAVQVIIGIVEDIAENH

54	P66937, DNA gyrase subunit B (gyrB)	7348	MVTALSDVNNTDNYGAGQIQVLEGLEAVRKRPGMYIGSTSERGLHHLVWEIVDNSIDEALAGYANKIEVVIEKDNWIKVTDNGRGIPVDIQEKMGRPAVEVILTVLHAGGKFGGGGYKVSGGLHGVGSSVVNALSQDLEVYVHRNETIYHQAYKKGVPQFDLKEVGTTDKTGTVIRFKADGEIFTETTVYNYETLQQRIRELAFLNKGIQITLRDERDEENVREDSYHYEGGIKSYVELLNENKEPIHDEPIYIHQSKDDIEVEIAIQYNSGYATNLLTYANNIHTYEGGTHEDGFKRALTRVLNSYGLSSKIMKEEKDRLSGEDTREGMTAIISIKHGDPQFEGQTKTKLGNSEVRQVVDKLFSEHFERFLYENPQVARTVVEKGIMAARARVAAKKAREVTRRKSALDVASLPGKLADCSSKSPEECEIFLVEGDSAGGSTKSGRDSRTQAILPLRGKILNVEKARLDRILNNNEIRQMITAFGTGIGGDFDLAKARYHKIVIMTDADVDGAHIRTLLLTFFYRFMRPLIEAGYVYIAQPPLYKLTQGKQKYYVYNDRELDKLKSELNPTPKWSIARYKGLGEMNADQLWETTMNPEHRALLQVKLEDAIEADQTFEMLMGDVVENRRQFIEDNAVYANLDF
55	A0A0H3JL12, SA0587 protein	3635	MKKLVPLLLALLLLVAACGTGGKQSSDKSNGKLKVVTTNSILYDMAKNVGGDNVDIHSIVPVGQDPHEYEVKPKDIKKLTDADVILYNGLNLETGNGWFEKALEQAGKSLKDKKVIAVSKDVKPIYLNGEEGNKDKQDPHAWLSLDNGIKYVKTIQQTFIDNDKKHKADYEKQGNKYIAQLEKLNNDSKDKFNDIPKEQRAMITSEGAFKYFSKQYGITPGYIWEINTEKQGTPEQMRQAIEFVKKHKLKHLLVETSVDKKAMESLSEETKKDIFGEVYTDSIGKEGTKGDSYYKMMKSNIETVHGSMK
56	A0A0H3JS82, Cassette chromosome recombinase B	3996	MQQLKTKRVGIYVRVSTEMQSTEGYSIDGQINQIKEYCDFHHFEVKDIYADRGISGKSMNRPELQRILKDAKEGYIDCVMVYKTNRLARNTSDLLKIVEDLHKQNVEFFSLSERMEVNTSSGKLMLQILASFSEFERNNIVENVFMGQTRRAQEGYYQGNLPLGYDKIPNSKHELMINQHEANIVKYIFESYAKGHGYRKIANALNHKGYVTKKGKPFSISSITYILANPFYIGKIQFAKYKDWSEKRRKGLNDKPVIAEGKHSPIINQDLWDKVQMRKKQVSQKPQVHGKGTNLLTGIIHCPQCGAPMAASNTTNTLKDGTKKRIRYYSCSNFRNKGSKVCSANSVRADVIEDYVMKQILEIVKSDKVIQRVVTHVNQENQVDGAALHHDIAYKQQQYDEVQIKLNNLIKTIEDNPDLTSVIRPSIQKYEKQLNDITNQINQLKNQQNEDKPLFDAKEISKLLQHIFHDIKHIEKSRLKALYLSVIDRIDIKKDGNHKKQFYVTLKLNNEIIKQLFNNKQLDEVHLSTSSLFLPQTLYLTI
57	A0A0H3JW57, SA2339 protein	11639	MAKFLYKMGTFIAKHKWSAVIAWIVIVAAILIPLATNAPKFDNDIKMTGLESLDTNKKIEKHFNQDSEKAQIRVVFKTTKDDGIVQPNITEDIKKTLEDIKKDDKHIDKISDPYENKQISKDKTTAFADITYDVSQTSLKDGSRDNVKSHLKDLRDNHNVQTELTGTGMTSTEVGGNSELVGIIVAFVVLLITFGSVIAAGLPIISALIGLASGVGIISLLTYAFDIPNVTLTLAVMIGLAVGIDYALFILFRYRQVMKTETDYIKGIGLAIGTAGSAVVFAGVTVVIAVCGLSLVGIDFLAVMGFASAISVIFAVFSALTLLPALISIFHKRIKVNKLQSNFKKDIDTPWSKFITGNALAAVLLGLIILVAAAIPVSHMRLGIPDDGVKPADSTQKKAYDIISDKFGEGFNGQIPMLINVKDKKDDPQGLQQDLQSVYKDIKDKKNVDIVTPPQMSKDNDYALMVVIPKQGPNAESTNDLVHDLRDYHKDAQDKYGFKTEISGQSVINIDMSKKLNEAIPLFATVIVVLAFFLLMIVFRSILIPLKAVLGFVLSLMATLGFTTLVMQDGFMKGLFGIETTGPMLAFLPVITIGILFGLAMDYEVFLMSRIHEEYSKTGDNDYSIKVGLKESGPVIVAAALIMFSVFFAFVFQEDVMIKSMGMALAFGVLFDAFVVRMMLIPALTKLFGKGSWYLPAWLNRIIPRVDIEGHALEKYKTVESQESEAKDSKETYDTTFKVYPQGATNVSKHQDVHGQDDAHSIVLDDKTMALYQEVKQQSASSLFLYDALIDYQNKHQLNSKQQVTNIEQLNKNIEKLNQLLEKNLRNKS

58	A0A0H3JNZ0, SA2437 protein	20300	MPKNKILIYLLSTTLVLPTLVSPTAYADTPQKDTTAKTTSHDSKKSNDDETSKDTTSKDTDKAD NNNTSNQDNNDKKFKTIDDSTSDSNNIIDFIYKNLPQTNINQLLTKNKYDDNYSLTTLIQNLFNL NSDISDYEQPRNGEKSTNDSNKNSDNSIKNDTDTQSSKQDKADNQKAPKSNNTKPSTSNKQPNS PKPTQPNQSNSQPASDDKANQKSSSKDNQSMSDSALDSILDQYSEDAKKTQKDYASQSKKDKN EKSNTKNPQLPTQDELKHKSKPAQSFNNDVNQKDTRATSLFETDPSISNNDDSGQFNVVDSKDT RQFVKSIAKDAHRIGQDNDIYASVMIAQAILESDSGRSALAKSPNHNLFGIKGAFEGNSVPFNTL EADGNKLYSINAGFRKYPSTKESLKDYSDLIKNGIDGNRTIYKPTWKSEADSYKDATSHLSKTY ATDPNYAKKLNSIIKHYQLTQFDDERMPDLDKYERSIKDYDDSSDEFKPFREVSDSMPYPHGQC TWYVYNRMKQFGTSISGDLGDAHNWNNRAQYRDYQVSHTPKRHAAVVFEAGQFGADQHYG HVAFVEKVNSDGSIVISESNVKGLGIISHRTINAAAAEELSYITGK
59		14918	MPKNKILIYLLSTTLVLPTLVSPTAYADTPQKDTTAKTTSHDSKKSNDDETSKDTTSKDTDKAD NNNTSNQDNNDKKFKTIDDSTSDSNNIIDFIYKNLPQTNINQLLTKNKYDDNYSLTTLIQNLFNL NSDISDYEQPRNGEKSTNDSNKNSDNSIKNDTDTQSSKQDKADNQKAPKSNNTKPSTSNKQPNS PKPTQPNQSNSQPASDDKANQKSSSKDNQSMSDSALDSILDQYSEDAKKTQKDYASQSKKDKN EKSNTKNPQLPTQDELKHKSKPAQSFNNDVNQKDTRATSLFETDPSISNNDDSGQFNVVDSKDT RQFVKSIAKDAHRIGQDNDIYASVMIAQAILESDSGRSALAKSPNHNLFGIKGAFEGNSVPFNTL EADGNKLYSINAGFRKYPSTKESLKDYSDLIKNGIDGNRTIYKPTWKSEADSYKDATSHLSKTY ATDPNYAKKLNSIIKHYQLTQFDDERMPDLDKYERSIKDYDDSSDEFKPFREVSDSMPYPHGQC TWYVYNRMKQFGTSISGDLGDAHNWNNRAQYRDYQVSHTPKRHAAVVFEAGQFGADQHYG HVAFVEKVNSDGSIVISESNVKGLGIISHRTINAAAAEELSYITGK
60		16019	MPKNKILIYLLSTTLVLPTLVSPTAYADTPQKDTTAKTTSHDSKKSNDDETSKDTTSKDTDKAD NNNTSNQDNNDKKFKTIDDSTSDSNNIIDFIYKNLPQTNINQLLTKNKYDDNYSLTTLIQNLFNL NSDISDYEQPRNGEKSTNDSNKNSDNSIKNDTDTQSSKQDKADNQKAPKSNNTKPSTSNKQPNS PKPTQPNQSNSQPASDDKANQKSSSKDNQSMSDSALDSILDQYSEDAKKTQKDYASQSKKDKN EKSNTKNPQLPTQDELKHKSKPAQSFNNDVNQKDTRATSLFETDPSISNNDDSGQFNVVDSKDT RQFVKSIAKDAHRIGQDNDIYASVMIAQAILESDSGRSALAKSPNHNLFGIKGAFEGNSVPFNTL EADGNKLYSINAGFRKYPSTKESLKDYSDLIKNGIDGNRTIYKPTWKSEADSYKDATSHLSKTY ATDPNYAKKLNSIIKHYQLTQFDDERMPDLDKYERSIKDYDDSSDEFKPFREVSDSMPYPHGQC TWYVYNRMKQFGTSISGDLGDAHNWNNRAQYRDYQVSHTPKRHAAVVFEAGQFGADQHYG HVAFVEKVNSDGSIVISESNVKGLGIISHRTINAAAAEELSYITGK
61	P61598, Putative surface protein SA2285	43354	MRDKKGPVNKRVDFLSNKLNKYSIRKFTVGTASILIGSLMYLGTQQEAEAAENNIENPTTLKDN VQSKEVKIEEVTNKDTAPQGVEAKSEVTSNKDTIEHEASVKAEDISKKEDTPKEVANVAEVQPK SSVTHNAEAPKVRKARSVDEGSFDITRDSKNVVESTPITIQGKEHFEGYGSVDIQKNPTDLGVSE VTRFNVGNESNGLIGALQLKNKIDFSKDFNFKVRVANNHQSNTTGADGWGFLFSKGNAEEYLT NGGILGDKGLVNSGGFKIDTGYIYTSSMDKTEKQAGQGYRGYGAFVKNDSSGNSQMVGENIDK SKTNFLNYADNSTNTSDGKFHGQRLNDVILTYVASTGKMRAEYAGKTWETSITDLGLSKNQAY NFLITSSQRWGLNQGINANGWMRTDLKGSEFTFTPEAPKTITELEKKLKRFHSRKNVNLIRI
62		23416	MRDKKGPVNKRVDFLSNKLNKYSIRKFTVGTASILIGSLMYLGTQQEAEAAENNIENPTTLKDN VQSKEVKIEEVTNKDTAPQGVEAKSEVTSNKDTIEHEASVKAEDISKKEDTPKEVANVAEVQPK SSVTHNAEAPKVRKARSVDEGSFDITRDSKNVVESTPITIQGKEHFEGYGSVDIQKNPTDLGVSE

			VTRFNVGNESNGLIGALQLKNKIDFSKDFNFKVRVANNHQSNTTGADGWGFLFSKGNAEEYLT NGGILGDKGLVNSGGFKIDTGYIYTSSMDKTEKQAGQGYRGYGAFVKNDSSGNSQMVGENIDK SKTNFLNYADNSTNTSDGKFHGQRLNDVILTYVASTGKMRAEYAGKTWETSITDLGLSKNQAY NFLITSSQRWGLNQGINANGWMRTDLKGSEFTFTPEAPKTITELEKKLKRFHSRKNVNLIRI
63		38919	MRDKKGPVNKRVDFLSNKLNKYSIRKFTVGTASILIGSLMYLGTQQEAEAAENNIENPTTLKDN VQSKEVKIEEVTNKDTAPQGVEAKSEVTSNKDTIEHEASVKAEDISKKEDTPKEVANVAEVQPK SSVTHNAEAPKVRKARSVDEGSFDITRDSKNVVESTPITIQGKEHFEGYGSVDIQKNPTDLGVSE VTRFNVGNESNGLIGALQLKNKIDFSKDFNFKVRVANNHQSNTTGADGWGFLFSKGNAEEYLT NGGILGDKGLVNSGGFKIDTGYIYTSSMDKTEKQAGQGYRGYGAFVKNDSSGNSQMVGENIDK SKTNFLNYADNSTNTSDGKFHGQRLNDVILTYVASTGKMRAEYAGKTWETSITDLGLSKNQAY NFLITSSQRWGLNQGINANGWMRTDLKGSEFTFTPEAPKTITELEKKLKRFHSRKNVNLIRI
64		6642.1	MRDKKGPVNKRVDFLSNKLNKYSIRKFTVGTASILIGSLMYLGTQQEAEAAENNIENPTTLKDN VQSKEVKIEEVTNKDTAPQGVEAKSEVTSNKDTIEHEASVKAEDISKKEDTPKEVA NVAEVQPKSSVTHNAEAPKVRKARSVDEGSFDITRDSKNVVESTPITIQGKEHFEGYGSVDIQKN PTDLGVSEVTRFNVGNESNGLIGALQLKNKIDFSKDFNFKVRVANNHQSNTTGADGWGFLFSK GNAEEYLTNGGILGDKGLVNSGGFKIDTGYIYTSSMDKTEKQAGQGYRGYGAFVKNDSSGNSQ MVGENIDKSKTNFLNYADNSTNTSDGKFHGQRLNDVILTYVASTGKMRAEYAGKTWETSITDL GLSKNQAYNFLITSSQRWGLNQGINANGWMRTDLKGSEFTFTPEAPKTITELEKKLKRFHSRKN VNLIRI
65		4484	MRDKKGPVNKRVDFLSNKLNKYSIRKFTVGTASILIGSLMYLGTQQEAEAAENNIENPTTLKDN VQSKEVKIEEVTNKDTAPQGVEAKSEVTSNKDTIEHEASVKAEDISKKEDTPKEVANVAEVQPK SSVTHNAEAPKVRKARSVDEGSFDITRDSKNVVESTPITIQGKEHFEGYGSVDIQKNPTDLGVSE VTRFNVGNESNGLIGALQLKNKIDFSKDFNFKVRVANNHQSNTTGADGWGFLFSKGNAEEYLT NGGILGDKGLVNSGGFKIDTGYIYTSSMDKTEKQAGQGYRGYGAFVKNDSSGNSQMVGENIDK SKTNFLNYADNSTNTSDGKFHGQRLNDVILTYVASTGKMRAEYAGKTWETSITDLGLSKNQAY NFLITSSQRWGLNQGINANGWMRTDLKGSEFTFTPEAPKTITELEKKLKRFHSRKNVNLIRI
66		2278	MRDKKGPVNKRVDFLSNKLNKYSIRKFTVGTASILIGSLMYLGTQQEAEAAENNIENPTTLKDN VQSKEVKIEEVTNKDTAPQGVEAKSEVTSNKDTIEHEASVKAEDISKKEDTPKEVANVAEVQPK SSVTHNAEAPKVRKARSVDEGSFDITRDSKNVVESTPITIQGKEHFEGYGSVDIQKNPTDLGVSE VTRFNVGNESNGLIGALQLKNKIDFSKDFNFKVRVANNHQSNTTGADGWGFLFSKGNAEEYLT NGGILGDKGLVNSGGFKIDTGYIYTSSMDKTEKQAGQGYRGYGAFVKNDSSGNSQMVGENIDK SKTNFLNYADNSTNTSDGKFHGQRLNDVILTYVASTGKMRAEYAGKTWETSITDLGLSKNQAY NFLITSSQRWGLNQGINANGWMRTDLKGSEFTFTPEAPKTITELEKKLKRFHSRKNVNLIRI
67	Q99SN7, Uncharacterized leukocidin-like protein 2	26908	MKNKKRVLIASSLSCAILLLSAATTQANSAHKDSQDQNKKEHVDKSQQKDKRNVTNKDKNST VPDDIGKNGKITKRTETVYDEKTNILQNLQFDFIDDPTYDKNVLLVKKQGSIHSNLKFESHKEEK NSNWLKYPSEYHVDFQVKRNRKTEILDQLPKNKISTAKVDSTFSYSSGGKFDSTKGIGRTSSNSY SKTISYNQQNYDTIASGKNNNWHVHWSVIANDLKYGGEVKNRNDELLFYRNTRIATVENPELS FASKYRYPALVRSGFNPEFLTYLSNEKSNEKTQFEVTYTRNQDILKNRPGIHYAPPILEKNKDGQ RLIVTYEVDWKNKTVKVVDKYSDDNKPYKEG

68		12206	MKNKKRVLIASSLSCAILLLSAATTQANSAHKDSQDQNKKEHVDKSQQKDKRNVTNKDKNST<br>VPDDIGKNGKITKRTETVYDEKTNILQNLQFDFIDDPTYDKNVLLVKKQGSIHSNLKFESHKEEK<br>NSNWLKYPSEYHVDFQVKRNRKTEILDQLPKNKISTAKVDSTFSYSSGGKFDSTKGIGRTSSNSY<br>SKTISYNQQNYDTIASGKNNNWHVHWSVIANDLKYGGEVKNRNDELLFYRNTRIATVENPELS<br>FASKYRYPALVRSGFNPEFLTYLSNEKSNEKTQFEVTYTRNQDILKNRPGIHYAPPILEKNKDGQ<br>RLIVTYEVDWKNKTVKVVDKYSDDNKPYKEG
69	P0A072, Gamma-hemolysin component A	10287	MIKNKILTATLAVGLIAPLANPFIEISKAENKIEDIGQGAEIIKRTQDITSKRLAITQNIQFDFVKDK<br>KYNKDALVVKMQGFISSRTTYSDLKKYPYIKRMIWPFQYNISLKTKDSNVDLINYLPKNKIDSA<br>DVSQKLGYNIGGNFQSAPSIGGSGSFNYSKTISYNQKNYVTEVESQNSKGVKWGVKANSFVTPN<br>GQVSAYDQYLFAQDPTGPAARDYFVPDNQLPPLIQSGFNPSFIT<br>TLSHERGKGDKSEFEITYGRNMDATYAYVTRHRLAVDRKHDAFKNRNVTVKYEVNWKTHE<br>VKIKSITPK
70		26908	MIKNKILTATLAVGLIAPLANPFIEISKAENKIEDIGQGAEIIKRTQDITSKRLAITQNIQFDFVKDK<br>KYNKDALVVKMQGFISSRTTYSDLKKYPYIKRMIWPFQYNISLKTKDSNVDLINYLPKNKIDSA<br>DVSQKLGYNIGGNFQSAPSIGGSGSFNYSKTISYNQKNYVTEVESQNSKGVKWGVKANSFVTPN<br>GQVSAYDQYLFAQDPTGPAARDYFVPDNQLPPLIQSGFNPSFITTLSHERGKGDKSEFEITYGRN<br>MDATYAYVTRHRLAVDRKHDAFKNRNVTVKYEVNWKTHE<br>VKIKSITPK
71	A0A0H3JLZ7, Probable L-asparaginase	12850	MKHLLVIHTGGTISMSQDQSNKVVTNDINPISMHQDVINQYAQIDELNPFNVPSPHMTIQHVKQ<br>LKDIILEAVTNKYYDGFVITHGTDTLEETAFLLDLILGIEQPVVITGAMRSSNEIGSDGLYNYISAI<br>RVASDEKARHKGVMVVFNDEIHTARNVTKTHTSNTNTFQSPNHGPLGVLTKDRVQFHHMPYR<br>QQALENVNDKLNVPLVKAYMGMPGDIFSFYSREGIDGMVIEALGQGNIPPSALEGIQQLVSLNIP<br>IVLVSRSFNGIVSPTYAYDGGGYQLAQQGFIFSNGSNGPKARLKLLVALSNNLDKAEIKSYFEL
72	A0A0H3JJ69, **YycI domain-containing protein**	13616	MNWKLTKTLFIFVFILVNIVLVSIYVNKVNRSHINEVESNNEVNFQQEEIKVPASILNKSVKGIKL<br>EQITGRSKDFSSKAKGDSDLTTSDGGKLLNANISQSVKVSDNNLKDLKDYVNKRVFKGSEYQLS<br>EINSGSVKYEQTYDNFPILNNSKAMLNFNIEDNKAASYKQSMMDDIKPTDGADKKHQVIGVRK<br>AIEALYYNRYLKKGDEVINARLGYYSVVNETNVQLLQPNWEIKVKHDGKDKTNTYYVEATNN<br>NPKIINH
73	Q7A3E0, 4,4'-diaponeurosporenoate glycosyltransferase	10118	MKWLSRILTVIVTMSMACGALIFNRRHQLKTKTLNFNHKALTIIIPARNEEKRIGHLLHSIIQQQV<br>PVDVIVMNDGSTDETARVARSYGATVVDVVDDTDGKWYGKSHACYQGVTHACTNRIAFVDA<br>DVTFLRKDAVETLINQYQLQGEKGLLSVQPYHITKRFYEGFSAIFNLMTVVGMNVFSTLDDGRT<br>NQHAFGPVTLTNKEDYYATGGHKSANRHIIEGFALGSAYTSQSLPVTVYEGFPFVAFRMYQEGF<br>QSLQEGWTKHLSTGAGGTKPKIMTAIVLWLFGSIASILGLCLSLKYRQMSVRKMVALYLSYTTQ<br>FIYLHRRVGQFSNLLMVCHPLLFMFFTKIFIQSWKQTHRYGVVEWKGRQYSISKEQ
74	A0A0H3JVS7, SA2164 protein	14483	MNIFKNKLLWIAPIATMIILVIFSLAFYPAYNPKPKDLPIGILNEDKGTTIQDKNVNIGKKLEDKLL<br>DSDSNKIKWVKVDSEKDLEKDLKDQKIFGVAIIDKDFSKDAMSKTQKVVMDSKKEEMQQKVA<br>SGEIPPQVVQQMKQKMGNQQVEVKQAKFKTIVSEGSSLQGSQIASAVLTGMGDNINAQITKQSL<br>ETLTSQNVKVNAADINGLTNPVKVDNEKLNKVKDHQAGGNAPFLMFMPIWIGSIVTSILLFFAF<br>RTSNNIVVQHRIIASIGQMIFAVVAAFAGSFVYIYFMQGVQGFDFDHPNRIAIFVAFAILGFVGLIL

			GVMVWLGMKSVPIFFILMFFSMQLVTLPKQMLPESYQKYVYDWNPFTHYATSVRELLYLNHHI ELNSTMWMFIGFMIFGAVSSLVSAIVRKHSTKRTEVPS
75	Q7A6L9, UPF0337 protein SA0772	6886	MADESKFEQAKGNVKETVGNVTDNKNLENEGKEDKASGKAKEFVENAKEKATDFIDKVKG NKGE
76	Q7A5P3, Cold shock protein CspA	7320	MKQGTVKWFNAEKGFGFIEVEGENDVFVHFSAINQDGYKSLEEGQAVEFEVVEGDRGPQAAN VVKL
77	P99154, 50S Ribosomal protein L7/L12	12578	MANHEQIIEAIKEMSVLELNDLVKAIEEEFGVTAAAPVAVAGAAGGADAAAEKTEFDVELTSA GSSKIKVVKAVKEATGLGLKDAKELVDGAPKVIKEALPKEEAEKLKEQLEEVGATVELK
78	P99122, Thioredoxin	11305.8	MAIVKVTDADFDSKVESGVQLVDFWATWCGPCKMIAPVLEELAADYEGKADILKLDVDEN PSTAAKYEVMSIPTLIVFKDGQPVDKVVGFQPKENLAEVLDKHL
79	Q7A5J1, DNA-binding protein HU	9626	MNKTDLINAVAEQADLTKKEAGSAVDAVFESIQNSLAKGEKVQLIGFGNFEVRERAARKGRNP QTGKEIDIPASKVPAFKAGKALKDAVK
80	P0A0G0, 30S ribosomal protein L30	6420	MAKLQITLTRSVIGRPETQRKTVEALGLKKTNSSVVVEDNPAIRGQINKVKHLVTVEEK
81	P66440, 30S ribosomal protein S16	10102	MAVKIRLTRLGSKRNPFYRIVVADARSPRDGRIIEQIGTYNPTSANAPEIKVDEALALKWLNDGA KPTDTVHNILSKEGIMKKFDEQKKAK
82	Q7A5C0, 30S ribosomal protein S20	8888	MANIKSAIKRVKTTEKAEARNISQKSAMRTAVKNAKTAVSNNADRIKSQLMTANKADNKNEL VSLAVKLVDKAAQSNLIHSNK
83	P60735, 50S ribosomal protein L24	11533	MHIKKGDNVKVIAGKDKGKEGKVIATLPKKDRVVVEGVNIMKKHQKPTQLNPEGGILETEAAI HVSNVQLLDPKTNEPTRVGYKFVDGKKVRIAKKSGEEIKSNN
84	P66494, 30S Ribosomal Protein S19	10525.3	MARSIKKGPFVDEHLMKKVEAQEGSEKKQVIKTWSRRSTIFPNFIGHTFAVYDGRKHVPVYVTE DMVGHKLGEFAPTRTFKGHVADDKKTRR
85	P66299, 50S Ribosomal Protein L36	3693	MKVRPSVKPICEKCKVIKRKGKVMVICENPKHKQRQG
86	P66276, 30S Ribosomal Protein L35	5292	MPKMKTHRGAAKRVKRTASGQLKRSRAFTSHLFANKSTKQKRQLRKARLVSKSDMKRVKQL LAYKK
87	P66210, 30S Ribosomal Protein L32	6349	MAVPKRRTSKTRKNKRRTHFKISVPGMTECPNCGEYKLSHRVCKNCGSYNGEEVAAK
88	P66521, 30S ribosomal protein S21	6840	MSKTVVRKNESLEDALRRFKRSVSKSGTIQEVRKREFYEKPSVKRKKKSEAARKRKFK
89	P99155, 50S ribosomal protein L10		MSAIIEAKKQLVDEIAEVLSNSVSTVIVDYRGLTVAEVTDLRSQLREAGVEYKVYKNTMVRRA AEKAGIEGLDEFLTGPTAIATSSEDAVAAAKVISGFAKDHEALEIKSGVMEGNVITAEEVKTVGS LPSHDGLVSMLLSVLQAPVRNFAYAVKAIGEQKEENAE
90	P66108, 50S ribosomal protein L20		MPRVKGGTVTRARRKKTIKLAKGYFGSKHTLYKVAKQQVMKSGQYAFRDRRQRKRDFRKLW ITRINAAARQHEMSYSRLMNGLKKAGIDINRKMLSEIAISDEKAFAQLVTKAKDALK
91	P66388, 30S ribosomal protein S13		MARIAGVDIPREKRVVISLTYIYGIGTSTAQKILEEANVSADTRVKDLTDDELGRIREVVDGYKV EGDLRRETNLNIKRLMEISSYRGIRHRRGLPVRGQKTKNNARTRKGPVKTVANKKK

92	Q7A5X8, 30S ribosomal protein S15		MAISQERKNEIIKEYRVHETDTGSPEVQIAVLTAEINAVNEHLRTHKKDHHSRRGLLKMVGRRR HLLNYLRSKDIQRYRELIKSLGIRR
93	Q7A593, UPF0337 protein SA1452	6551	MADESKFDQFKGNVKETVGNVTDNKELEKEGQQDKATGKAKEVVENAKNKITDAIDKLKK
94	P66173, 50S Ribosomal Protein L29	8090	MKAKEIRDLTTSEIEEQIKSSKEELFNLRFQLATGQLEETARIRTVRKTIARLKTVAREREIEQSKA NQ
95	P66196, 50S Ribosomal Protein L31 Type B	9721	MKQGIHPEYHQVIFLDTTTNFKFLSGSTKTSSEMMEWEDGKEYPVIRLDISSDSHPFYTGRQKFA AADGRVERFNKKFGLKSNN
96	P66726, DNA-directed RNA polymerase subunit omega	8149	MLNPPLNQLTSQIKSKYLIATTAAKRAREIDEQPETELLSEYHSFKPVGRALEEIADGKIRPVISSD YYGKE
97	P99132, Probable tautomerase SA1195.1	6611.5	MMPIVNVKLLEGRSDEQLKNLVSEVTDAVEKTTGANRQAIHVVIEEMKPNHYGVAGVRKSDQ
98		6626(-14Da modification)	MPIVNVKLLEGRSDEQLKNLVSEVTDAVEKTTGANRQAIHVVIEEMKPNHYGVAGVRKSDQ
99	A0A0H3JV47,Uncharacterized protein	8211	METKYELNNTKKVANAFGLNEADTNLLINAVDLDIKNNMQEISSELQQSEQSKQKQYGTTLQN LAKQNRIIK
100	Q7A473, 50S ribosomal protein L13	16332	MRQTFMANESNIERKWYVIDAEGQTLGRLSSEVASILRGKNKVTYTPHVDTGDYVIVINASKIE FTGNKETDKVYYRHSNHPGGIKSITAGELRRTNPERLIENSIKGMLPSTRLGEKQG KKLFVYGGAEHPHAAQQPENYELRG
101	Q7A460, 50S ribosomal protein L22	12833	MEAKAVARTIRIAPRKVRLVLDLIRGKNAAEAIAILKLTNKASSPVIEKVLMSALANAEHNYDM NTDELVVKEAYANEGPTLKRFRPRAQGRASAINKRTSHITIVVSDGKEEAKEA
102	P99142, 30S ribosomal protein S6	11594	MRTYEVMYIVRPNIEEDAKKALVERFNGILATEGAEVLEAKDWGKRRLAYEINDFKDGFYNIV RVKSDNNKATDEFQRLAKISDDIIRYMVIREDEDK
103	P66334, 30S ribosomal protein S10	11444	MAKQKIRIRLKAYDHRVIDQSAEKIVETAKRSGADVSGPIPLPTEKSVYTIIRAVHKYKDSREQF EQRTHKRLIDIVNPTPKTVDALMGLNLPSGVDIEIKL
104	P68800, Fibrinogen-binding protein (Efb)	11104	MKNKLIAKSLLTIAAIGITTTTIASTADASEGYGPREKKPVSINHNIVEYNDGTFKYQSRPKFNST PKYIKFKHDYNILEFNDGTFEYGARPQFNKPAAKTDATIKKEQKLIQAQNLVREFEKTHTVSAH RKAQKAVNLVSFEYKVKKMVLQERIDNVLKQGLVR
105	A0A0H3JPH2, Uncharacterized protein	3180	MKKKFVSSCIASTILFGTLLGVTYKAEAATVHVAGGVWSHGIGKHYVWSYYSHNKRNHGSTA VGKYSSFSGVARPGVQSKASAPKAWGGNKTFYSLH
106	Hypothetical protein	8620	MKKKFVSSCIASTILFGTLLGVTYKAEAATVHVAGGVWSHGIGKHYVWSYYSHNKRNHGSTA VGKYSSFSGVARPGVQSKASAPKAWGGNKTFYSLH
107	Hypothetical protein	8769	MKKKFVSSCIASTILFGTLLGVTYKAEAATVHVAGGVWSHGIGKHYVWSYYSHNKRNHGSTA VGKYSSFSGVARPGVQSKASAPKAWGGNKTFYSLH
108	Hypothetical protein	8239	MKKKFVSSCIASTILFGTLLGVTYKAEAATVHVAGGVWSHGIGKHYVWSYYSHNKRNHGSTA VGKYSSFSGVARPGVQSKASAPKAWGGNKTFYSLH
109		5457	MKKKFVSSCIASTILFGTLLGVTYKAEAATVHVAGGVWSHGIGKHYVWSYYSHNKRNHGSTA VGKYSSFSGVARPGVQSKASAPKAWGGNKTFYSLH

110	A0A0H3JLH5, Uncharacterized protein	7660	MKFKKVLVATAMVGVLATGVVGYGNQADAKVYSQNGLVLHDDANFLEHELSYIDVLLDKNA DQATKDNLRSYFADKGLHSIKDIINKAKQDGFDVSKYEHVK
111	A0A0H3JVL1, SA2097 protein	15003	MKKLVTATTLTAGIGTALVGQAHHADAAENYTNYNNYNYNTTQTTTTTTTTTTSSISHSGNL YTAGQCTWYVYDKVGGEIGSTWGNANNWAAAAQGAGFTVNHTPSKGAILQSSEGPFGHVAY VESVNSDGSVTISEMNYSGGPFSVSSRTISASEAGNYNYIHI
112	P65415, Putative 3-methyladenine DNA glycosylase	4208	MDFVNNDTRQIAKNLLGVKVIYQDTTQTYTGYIVETEAYLGLNDRAAHGYGGKITPKVTSLYK RGGTIYAHVMHTHLLINFVTKSEGIPEGVLIRAIEPEEGLSAMFRNRGKKGYEVTNGPGKWTKA FNIPRAIDGATLNDCRLSIDTKNRKYPKDIIASPRIGIPNKGDWTHKSLRYTVKGNPFVSRMRKSD CMFPEDTWK
113	Q99RL2, IgG-binding protein SBI	5285	MKNKYISKLLVGAATITLATMISNGEAKASENTQQTSTKHQTTQNNYVTDQQKAFYQVLHLKG ITEEQRNQYIKTLREHPERAQEVFSESLKDSKNPDRRVAQQNAFYNVLKNDNLTEQEKNNYIAQ IKENPDRSQQVWVESVQSSKAKERQNIENADKAIKDFQDNKAPHDKSAAYEANSKLPKDLRDK NNRFVEKVSIEKAIVRHDERVKSANDAISKLNEKDSIENRRLAQREVNKAPMDVKEHLQKQLD ALVAQKDAEKKVAPKVEAPQIQSPQIEKPKAESPKVEVPQIQSPKVEVPQSKLLGYYQSLKDSF NYGYKYLTDTYKSYKEKYDTAKYYYNTYYKYKGAIDQTVLTVLGSGSKSYIQPLKVDDKNGY LAKSYAQVRNYVTESINTGKVLYTFYQNPTLVKTAIKAQETASSIKNTLSNLLSFWK
114		4007	MKNKYISKLLVGAATITLATMISNGEAKASENTQQTSTKHQTTQNNYVTDQQKAFYQVLHLKG ITEEQRNQYIKTLREHPERAQEVFSESLKDSKNPDRRVAQQNAFYNVLKNDNLTEQEKNNYIAQ IKENPDRSQQVWVESVQSSKAKERQNIENADKAIKDFQDNKAPHDKSAAYEANSKLPKDLRDK NNRFVEKVSIEKAIVRHDERVKSANDAISKLNEKDSIENRRLAQREVNKAPMDVKEHLQKQLD ALVAQKDAEKKVAPKVEAPQIQSPQIEKPKAESPKVEVPQIQSPKVEVPQSKLLGYYQSLKDSF NYGYKYLTDTYKSYKEKYDTAKYYYNTYYKYKGAIDQTVLTVLGSGSKSYIQPLKVDDKNGY LAKSYAQVRNYVTESINTGKVLYTFYQNPTLVKTAIKAQETASSIKNTLSNLLSFWK
115	A0A0H3JLA2, Uncharacterized protein	10613	MKPYIQLVLFKQWLQYILLVTTIVIALVLIGIGYRVAHDNFKIPITIQDLDQTTASKSFVNKIKQSD YVTIKKVDEDESYIEDDVTKKEAILSMQIPKGFSQKLKENRLKETIQLYGRDDFIGGIAIEIVSSSL YEQQIPNIIYEHLEDMKQHQSIDAINKSYHKHTPESKIKFVSLTKQAQHSISISLIFAVILFVSAVQV VLHYRLNQQAALQRLSQYHLSRFKLYSTYVMTHTILLLLVLLAVSLYLSQPLSLIFYLKSLLLILI YEIGIVFILFHIQTISHRLFMTFIYALAMGIVYLIIFM
116	Q99SU8, Chemotaxis inhibitory protein	14127	MKKKLATTVLALSFLTAGISTHHHSAKAFTFEPFPTNEEIESNKKMLEKEKAYKESFKNSGLPTT LGKLDERLRNYLEKGTKNTAQFEKMVILTENKGYYTVYLNTPLAEDRKNVELLGKMYKTYFF KKGESKSSYVINGPGKTNEYAY
117		10920	MKKKLATTVLALSFLTAGISTHHHSAKAFTFEPFPTNEEIESNKKMLEKEKAYKESFKNSGLPTT LGKLDERLRNYLEKGTKNTAQFEKMVILTENKGYYTVYLNTPLAEDRKNVELLGKMYKTYFF KKGESKSSYVINGPGKTNEYAY
118	A0A0H3JPQ1, SA1000 protein	10478	MKKNFIGKSILSIAAISLTVSTFAGESHAQTKNVEAAKKYDQYQTNFKKQVNKKVVDAQKAVN LFKRTRTVATHRKAQRAVNLIHFQHSYEKKKLQRQIDLVLKYNTLK
119	A0A0H3JVY4, Probable glycine betaine/ carnitine/	3995	MIEFLHEHGGQLMSKTLEHFYISIVALLLAIIVAVPIGILLSKTKRTANIVLTVAGVLQTIPTLAVL AIMIPIFGVGKTPAIVALFIYVLLPILNNTVLGVQNIDSNIKEAGKSMGMTQFQLMKDVELPLALP

	choline ABC transporter opuCB		LIIGGIRLSSVYVISWATLASYVGAGGLGDFIFNGLNLYDPLMIVTATVLVTALALGVDALLALV EKWVVPKGLKVSG
120	0A0H3JMB8, Citrate synthase	12595	MAELQRGLEGVIAAETKISSIIESQLTYAGYDIDDLAENAQFEEVIFLLWNYRLPNEEELAHLKG KLNQYMTLNPRVYTHFEEYVTDHVHPMTALRTSLSYIAHFDPDAENESDENRYERAMRIQAKV ASLVTAFARVRQDKEPLKPNPDLSYAANFLYMLRGELPTDIEVEAFNKALILHADHELNASAFT ARCAVSSLSDMYSGIVAAVGSLKGPLHGGANEQVMTMLSEIGSIENVDAYLDEKFANKDKVM GFGHRVYKDGDPRAKYLREMSRQITKDAGREELFEMSVKMEKRMAEEKGLIPNVDFYSASVY HCMEIPHDLFTPIFAVSRSAGWIAHILEQYKDNRIMRPRAKYIGETNRKYIPLEERK
121	A0A0H3JK15, Uncharacterized protein	17424	MKKLLTASIIACSVVMGVGLVNTSAEAASGNSIDTVKQLIKGDQSLENVKIGESIKDVLTKYKNP MYSYNEDGTEHYYEFHTKKGMLLVTTDGKKNNGKVTHISMMYNDANGPTYQAVKNYVGKA VTHTEYSKVAGNFGYIEKGKTTYQFASAPKDKNIKLYRIDLEK
122		17321	MKKLLTASIIACSVVMGVGLVNTSAEAASGNSIDTVKQLIKGDQSLENVKIGESIKDVLTKYKNP MYSYNEDGTEHYYEFHTKKGMLLVTTDGKKNNGKVTHISMMYNDANGPTYQAVKNYVGKA VTHTEYSKVAGNFGYIEKGKTTYQFASAPKDKNIKLYRIDLEK
123		16847	MKKLLTASIIACSVVMGVGLVNTSAEAASGNSIDTVKQLIKGDQSLENVKIGESIKDVLTKYKNP MYSYNEDGTEHYYEFHTKKGMLLVTTDGKKNNGKVTHISMMYNDANGPTYQAVKNYVGKA VTHTEYSKVAGNFGYIEKGKTTYQFASAPKDKNIKLYRIDLEK
124	A0A0H3JTC5, DM13 domain-containing protein	11668	MNTKYFLAVGAVASVLTLGACGNSNSQDQGNKTEQKTKSEDSNVKTDKTKHLTGTFSSKNGE TVEGKAEIKNGKLMLTNYKSSKGPDLYVYLTKNGDIKNGKEIAMVDYDKEKQTFDLKNVDLS KYDEVTIYCKKAHVIFGGAKLK
125		15146	MNTKYFLAVGAVASVLTLGACGNSNSQDQGNKTEQKTKSEDSNVKTDKTKHLTGTFSSKNGE TVEGKAEIKNGKLMLTNYKSSKGPDLYVYLTKNGDIKNGKEIAMVDYDKEKQTFDLKNVDLS KYDEVTIYCKKAHVIFGGAKLK
126		12894	MNTKYFLAVGAVASVLTLGACGNSNSQDQGNKTEQKTKSEDSNVKTDKTKHLTGTFSSKNGE TVEGKAEIKNGKLMLTNYKSSKGPDLYVYLTKNGDIKNGKEIAMVDYDKEKQTFDLKNVDLS KYDEVTIYCKKAHVIFGGAKLK
127	A0A0H3JMR5, Uncharacterized protein	10570	MALFLYKNLRRSFNMANEIIKKTERFILVQIDKEGTERVLYQDFVGSFTTSDSASYAQDFKSEEN AKKIAETLNLLYQLTGNQNGVKVVKEVVDRTDLSSDKSVDSETM
128	Q7A377, Immunodominant staphylococcal antigen B	18565	MNKTSKVCVAATLALGTLIGVTVVENSAPTSKQAQAAITPYYTYNGYIGNNANFILDKNFINAI KYDNVKFNGIKLAKTNTIKKVEKYDQTFKGVSAKGNEASQLQFVVKNNISLKDIQKAYGKDLK KENGKTKEADSGIFYYQNAKKTLGIWFVVDHNRVVEVTVGHTPYKTSK
129		17837	MNKTSKVCVAATLALGTLIGVTVVENSAPTSKQAQAAITPYYTYNGYIGNNANFILDKNFINAI KYDNVKFNGIKLAKTNTIKKVEKYDQTFKGVSAKGNEASQLQFVVKNNISLKDIQKAYGKDLK KENGKTKEADSGIFYYQNAKKTLGIWFVVDHNRVVEVTVGHTPYKTSK
130		14699	MNKTSKVCVAATLALGTLIGVTVVENSAPTSKQAQAAITPYYTYNGYIGNNANFILDKNFINAI KYDNVKFNGIKLAKTNTIKKVEKYDQTFKGVSAKGNEASQLQFVVKNNISLKDIQKAYGKDLK KENGKTKEADSGIFYYQNAKKTLGIWFVVDHNRVVEVTVGHTPYKTSK
131		16575	MNKTSKVCVAATLALGTLIGVTVVENSAPTSKQAQAAITPYYTYNGYIGNNANFILDKNFINAI KYDNVKFNGIKLAKTNTIKKVEKYDQTFKGVSAKGNEASQLQFVVKNNISLKDIQKAYGKDLK KENGKTKEADSGIFYYQNAKKTLGIWFVVDHNRVVEVTVGHTPYKTSK

132	A0A0H3JMD3, Uncharacterized protein	3448(-14Da)	MDINVLATIFKFILFVVEIYYFGMIIYFFTSWVPSIRETKVGYFLAKIYEPFLQPFRKVIPPIGIIDISS IAAIIVLVLFQKGLLQIFNWILIQLQ
133	Q99SU9, Staphylococcal complement inhibitor	9790	MKIRKSILAGTLAIVLASPLVTNLDKNEAQASTSLPTSNEYQNEKLANELKSLLDELNVNELATG SLNTYYKRTIKISGLKAMYALKSKDFKKMSEAKYQLQKIYNEIDEALKSKY
134		4133	MKIRKSILAGTLAIVLASPLVTNLDKNEAQASTSLPTSNEYQNEKLANELKSLLDELNVNELATG SLNTYYKRTIKISGLKAMYALKSKDFKKMSEAKYQLQKIYNEIDEALKSKY
135		9806	MKIRKSILAGTLAIVLASPLVTNLDKNEAQASTSLPTSNEYQNEKLANELKSLLDELNVNELATG SLNTYYKRTIKISGLKAMYALKSKDFKKMSEAKYQLQKIYNEIDEALKSKY
136		4133	MKIRKSILAGTLAIVLASPLVTNLDKNEAQASTSLPTSNEYQNEKLANELKSLLDELNVNELATG SLNTYYKRTIKISGLKAMYALKSKDFKKMSEAKYQLQKIYNEIDEALKSKY
137		9595.9	MKIRKSILAGTLAIVLASPLVTNLDKNEAQASTSLPTSNEYQNEKLANELKSLLDELNVNELATG SLNTYYKRTIKISGLKAMYALKSKDFKKMSEAKYQLQKIYNEIDEALKSKY
